# Supplementary material for: Deviation in development of dorsal association tracts during preadolescence links to concurrent and future cognitive performance and transdiagnostic psychopathology
Source: Nat Commun. 2026 Feb 19;17:2943. doi: 10.1038/s41467-026-69774-6 (PMC13031853; doi:10.1038/s41467-026-69774-6)
Supplement: Supplementary file 1 — Supplementary Information [file 41467_2026_69774_MOESM1_ESM.pdf]

## Supplementary Material

**Supplementary Table 1. Names, abbreviations and mainly connected brain regions (DK atlas) of the included white matter (WM) tracts**

| Tract Name | Abbrev. | Region 1 | Region 2 |
|---|---|---|---|
| Arcuate Fasciculus | AF | Superior temporal | Pars opercularis |
| Frontal Aslant Tract | FAT | Superior frontal | Pars opercularis |
| Parietal Aslant Tract | PAT | Inferior parietal | Middle temporal |
| Superior Longitudinal Fasciculus 1 | SLF1 | Superior frontal | Precuneus |
| Superior Longitudinal Fasciculus 2 | SLF2 | Inferior parietal | Precentral |
| Superior Longitudinal Fasciculus 3 | SLF3 | Inferior parietal | Pars opercularis |
| Inferior Fronto Occipital Fasciculus | IFOF | Lateral orbitofrontal | Lateral occipital |
| Inferior Longitudinal Fasciculus | ILF | Lateral occipital | Superior temporal |
| Uncinate Fasciculus | UF | Superior temporal | Lateral orbitofrontal |
| Middle Longitudinal Fasciculus | MdLF | Superior temporal | Middle temporal |
| Cingulum Frontal Parahippocampal | C_FPH | Isthmus cingulate | Superior frontal |
| Cingulum Frontal Parietal | C_FP | Precuneus | Rostral anterior cingulate |
| Cingulum Parahippocampal | C_PH | Isthmus cingulate | Inferior temporal |
| Cingulum Parahippocampal Parietal | C_PHP | Entorhinal | Precuneus |
| Cingulum Parolfactory | C_PO | Medial orbitofrontal | Precuneus |
| Fornix | F | Mammillary body | Hippocampus |
| Corticospinal Tract | CST | Precentral | Brainstem |
| Optic Radiation | OR | Lateral occipital | Thalamus |
| Vertical Occipital Fasciculus | VOF | Superior parietal | Lateral occipital |
| Thalamic Radiation Anterior | TR_A | Thalamus | Lateral orbitofrontal |
| Thalamic Radiation Posterior | TR_P | Thalamus | Lateral occipital |
| Thalamic Radiation Superior | TR_S | Thalamus | Superior parietal |
| Corticostriatal Tract Anterior | CS_A | Striatum | Lateral orbitofrontal/ Superior frontal |
| Corticostriatal Tract Posterior | CS_P | Striatum | Pericalcarine/Inferior_parietal/ Lateral occipital |
| Corticostriatal Tract Superior | CS_S | Striatum | Superior parietal/ Precentral/Postcentral/Superiorfrontal/Paracentral/Postcentral |
| Corpus Callosum Body | CC_Body | Superior frontal/Paracentral/Postcentral/Superior parietal | |
| Corpus Callosum Forceps Major | CC_Major | Cuneus/Superior parietal | |
| Corpus Callosum Forceps Minor | CC_Minor | Frontalpole/Superior frontal/Medial orbitofrontal | |
| Corpus Callosum Tapetum | CC_Tap | Hippocampus | |

Notes: the connected cortical regions are defined in the Desikan-Killiany (DK) atlas[1].

**Supplementary Table 2.** **Five-fold cross-validation performance of prediction models in HCP-D dataset**

| Tract | Uncorrected | | Corrected | |
|---|---|---|---|---|
| | *R* | RMSE (years) | *R* | RMSE (years) |
| Whole-brain | 0.855 | 2.264 | 0.935 | 1.574 |
| AF | 0.663 | 3.135 | 0.905 | 1.955 |
| C_FPH | 0.662 | 3.048 | 0.906 | 1.924 |
| C_FP | 0.639 | 3.147 | 0.901 | 1.938 |
| C_PH | 0.631 | 3.201 | 0.907 | 1.945 |
| C_PHP | 0.658 | 3.138 | 0.907 | 1.944 |
| C_PO | 0.537 | 3.454 | 0.919 | 1.761 |
| CST | 0.742 | 2.850 | 0.909 | 1.953 |
| CS_A | 0.619 | 3.239 | 0.915 | 1.871 |
| CS_P | 0.777 | 2.693 | 0.912 | 1.943 |
| CS_S | 0.738 | 2.825 | 0.905 | 1.973 |
| F | 0.497 | 3.521 | 0.925 | 1.710 |
| FAT | 0.763 | 2.746 | 0.908 | 1.957 |
| IFOF | 0.693 | 3.036 | 0.908 | 1.911 |
| ILF | 0.662 | 3.137 | 0.904 | 1.951 |
| MdLF | 0.693 | 3.017 | 0.908 | 1.915 |
| OR | 0.646 | 3.148 | 0.909 | 1.912 |
| PAT | 0.781 | 2.633 | 0.912 | 1.904 |
| SLF1 | 0.652 | 3.085 | 0.906 | 1.945 |
| SLF2 | 0.727 | 2.864 | 0.905 | 1.959 |
| SLF3 | 0.724 | 2.942 | 0.913 | 1.907 |
| TR_A | 0.731 | 2.803 | 0.909 | 1.885 |
| TR_P | 0.755 | 2.720 | 0.908 | 1.944 |
| TR_S | 0.728 | 2.808 | 0.910 | 1.903 |
| UF | 0.511 | 3.515 | 0.927 | 1.711 |
| VOF | 0.675 | 3.060 | 0.907 | 1.944 |
| CC_Body | 0.780 | 2.650 | 0.910 | 1.957 |
| CC_Major | 0.681 | 3.041 | 0.908 | 1.967 |
| CC_Minor | 0.411 | 3.687 | 0.936 | 1.528 |
| CC_Tap | 0.602 | 3.267 | 0.908 | 1.890 |

**Supplementary Table 3. Testing performance of brain age prediction models in HBN dataset**

| Tract | Uncorrected | | Corrected | |
|---|---|---|---|---|
| | R | RMSE (years) | R | RMSE (years) |
| Whole-brain | 0.622 | 3.337 | 0.935 | 2.027 |
| AF | 0.230 | 4.522 | 0.905 | 2.600 |
| C_FPH | 0.374 | 4.165 | 0.906 | 2.652 |
| C_FP | 0.385 | 4.019 | 0.901 | 2.062 |
| C_PH | 0.320 | 4.152 | 0.907 | 2.007 |
| C_PHP | 0.448 | 3.898 | 0.907 | 1.976 |
| C_PO | 0.205 | 4.555 | 0.919 | 1.864 |
| CST | 0.335 | 4.326 | 0.909 | 2.734 |
| CS_A | 0.301 | 5.313 | 0.915 | 4.461 |
| CS_P | 0.467 | 3.773 | 0.912 | 2.328 |
| CS_S | 0.548 | 3.506 | 0.905 | 2.436 |
| F | 0.290 | 4.400 | 0.925 | 1.719 |
| FAT | 0.547 | 3.514 | 0.908 | 2.371 |
| IFOF | 0.370 | 4.176 | 0.908 | 2.497 |
| ILF | 0.328 | 4.266 | 0.904 | 2.273 |
| MdLF | 0.313 | 4.321 | 0.908 | 2.512 |
| OR | 0.542 | 3.738 | 0.909 | 1.883 |
| PAT | 0.074 | 4.974 | 0.912 | 3.374 |
| SLF1 | 0.357 | 4.125 | 0.906 | 2.267 |
| SLF2 | 0.379 | 4.212 | 0.905 | 2.960 |
| SLF3 | 0.152 | 4.850 | 0.913 | 3.038 |
| TR_A | 0.461 | 3.740 | 0.909 | 2.452 |
| TR_P | 0.559 | 3.609 | 0.908 | 2.055 |
| TR_S | 0.437 | 4.023 | 0.910 | 2.988 |
| UF | 0.122 | 4.752 | 0.927 | 2.256 |
| VOF | 0.259 | 4.414 | 0.907 | 2.328 |
| CC_Body | 0.585 | 3.528 | 0.804 | 2.500 |
| CC_Major | 0.456 | 4.014 | 0.826 | 2.175 |
| CC_Minor | 0.151 | 4.835 | 0.828 | 2.067 |
| CC_Tap | 0.539 | 3.759 | 0.883 | 1.739 |

**Supplementary Table 4. Testing performance of brain age prediction models in independent ABCD dataset**

| Tract | *R* | RMSE (years) | *R* | RMSE (years) |
|---|---|---|---|---|
| Whole-brain | 0.543 | 2.041 | 0.699 | 1.620 |
| AF | 0.283 | 3.245 | 0.529 | 2.679 |
| C_FPH | 0.355 | 2.884 | 0.570 | 2.297 |
| C_FP | 0.395 | 2.637 | 0.631 | 2.002 |
| C_PH | 0.346 | 2.828 | 0.642 | 1.913 |
| C_PHP | 0.354 | 2.757 | 0.638 | 1.899 |
| C_PO | 0.255 | 3.121 | 0.637 | 1.891 |
| CST | 0.405 | 2.608 | 0.631 | 2.138 |
| CS_A | 0.188 | 4.685 | 0.352 | 4.333 |
| CS_P | 0.435 | 2.500 | 0.611 | 2.117 |
| CS_S | 0.426 | 2.538 | 0.602 | 2.101 |
| F | 0.258 | 3.046 | 0.653 | 1.766 |
| FAT | 0.302 | 3.253 | 0.500 | 2.941 |
| IFOF | 0.363 | 2.718 | 0.603 | 2.180 |
| ILF | 0.325 | 3.046 | 0.547 | 2.582 |
| MdLF | 0.305 | 3.048 | 0.579 | 2.370 |
| OR | 0.338 | 2.858 | 0.636 | 1.896 |
| PAT | 0.187 | 4.349 | 0.403 | 3.858 |
| SLF1 | 0.381 | 2.799 | 0.581 | 2.359 |
| SLF2 | 0.283 | 4.239 | 0.396 | 4.149 |
| SLF3 | 0.238 | 4.165 | 0.400 | 3.888 |
| TR_A | 0.431 | 2.599 | 0.569 | 2.294 |
| TR_P | 0.369 | 2.799 | 0.581 | 2.311 |
| TR_S | 0.298 | 3.486 | 0.418 | 3.227 |
| UF | 0.237 | 3.480 | 0.515 | 2.650 |
| VOF | 0.158 | 4.072 | 0.386 | 3.494 |
| CC_Body | 0.368 | 3.043 | 0.517 | 2.873 |
| CC_Major | 0.323 | 3.384 | 0.453 | 3.190 |
| CC_Minor | 0.156 | 3.553 | 0.608 | 2.092 |
| CC_Tap | 0.289 | 2.973 | 0.651 | 1.767 |

**Supplementary Table 5. Comparisons of loading distributions from two sCCA modes derived from bootstrap resampling tests (N = 1,000)**

| Behavioral measure | T | Effect size (Cohen's *d*) |
|---|---|---|
| CBCL_syn_social_prob | 3.770 | 0.158 |
| **CBCL_syn_attention_prob** | **-16.773** | **0.927** |
| **CBCL_total** | **-25.241** | **1.387** |
| **CBCL_dsm_ADHD** | **-23.537** | **1.269** |
| **PGBI_mania_total** | **24.463** | **0.920** |
| nihtbx_pic_vocab | -3.117 | 0.205 |
| nihtbx_flanker | -7.195 | 0.332 |
| nihtbx_list_sort | 6.543 | 0.379 |
| **nihtbx_card_sort** | **-10.869** | **0.668** |
| **nihtbx_pattern_comp** | -17.167 | **1.010** |
| nihtbx_pic_seq_memory | -6.261 | 0.264 |
| nihtbx_oral_reading | 3.089 | 0.185 |
| nihtbx_fluid_comp | -0.725 | 0.053 |
| nihtbx_cryst_comp | 1.859 | 0.130 |
| nihtbx_total_comp | 1.627 | 0.124 |
| **little_man_efficiency** | **-20.694** | **1.074** |
| matrix_reasoning | 1.558 | 0.086 |
| verbal_learning_immediate | -6.073 | 0.234 |
| verbal_learning_delayed | -0.991 | 0.035 |
| **SST_ssrt** | **28.713** | **0.977** |
| X2_back | -1.569 | 0.091 |
| recog_memory | -1.024 | 0.049 |

**Notes**: Behavioral measures showing significant differences in loading distributions between the two sCCA modes (effect size > 0.5) are highlighted.

**Supplementary Table 6. Selected Neurosynth topics and their abbreviations**

| Abbreviation | Topic |
|---|---|
| Attention | 49_attention_attentional_task |
| Cognitive-control and task performance | 11_task_cognitive_performance |
| Cognitive task demands | 36_task_difficulty_demands |
| Numerical Representation and Processing | 18_number_numerical_numbers |
| Representation | 55_representations_representation_abstract |
| Learning and training | 48_learning_training_sequence |
| Language | 12_language_comprehension_sentences |
| Reading and words | 21_reading_words_word |
| Working memory | 45_memory_working_wm |
| Mental imagery | 9_imagery_mental_events |
| Cognitive Conflict | 14_conflict_interference_control |
| Response inhibition | 89_inhibition_response_control |
| Contextual Modulation and Influence | 42_context_influence_modulation |
| Speech | 31_speech_auditory_production |
| Perception | 50_perceptual_perception_interaction |
| Semantic words | 23_semantic_words_word |
| Schizophrenia and Psychotic Symptoms | 10_schizophrenia_symptoms_risk |
| Social Cognition and Empathy | 92_social_empathy_moral |
| Recognition Memory | 76_memory_recognition_items |
| Memory Encoding | 72_memory_encoding_hippocampus |
| Reasoning | 71_reasoning_mind_mental |
| Emotion Regulation | 35_regulation_emotion_reappraisal |
| Personality Traits | 58_personality_trait_traits |
| Decision making | 53_decision_making_risk |
| Episodic Memory | 41_memory_retrieval_episodic |
| Emotion and Affective Evaluation | 30_emotional_negative_positive |
| Depression | 5_depression_mdd_disorder |
| ADHD and Eating Disorders | 54_adhd_food_weight |
| Substance Abuse and Stress Response | 75_stress_alcohol_users |
| Fear Conditioning and PTSD | 26_fear_anxiety_ptsd |
| Reward | 33_reward_striatum_ventral |

**Supplementary Table 7. Functional decoding results for selected Neurosynth topics. Significant results are bolded for each tract.**

| Topic | Mode 1 | | Mode 2 | |
|---|---|---|---|---|
| | rho | $p_{FDR}$ | rho | $p_{FDR}$ |
| Depression | -0.547 | 1.000 | 0.543 | **0.037** |
| Mental imagery | 0.432 | 0.093 | -0.240 | 1.000 |
| Schizophrenia and Psychotic Symptoms | -0.138 | 1.000 | -0.033 | 1.000 |
| Cognitive-control and task performance | 0.536 | **0.037** | -0.475 | 1.000 |
| Language | 0.549 | **0.037** | -0.666 | 1.000 |
| Cognitive Conflict | 0.387 | 0.158 | -0.351 | 1.000 |
| Numerical Representation and Processing | 0.522 | **0.043** | -0.397 | 1.000 |
| Reading and Words | 0.600 | **0.037** | -0.673 | 1.000 |
| Semantic words | 0.466 | 0.053 | -0.495 | 1.000 |
| Fear Conditioning and PTSD | -0.532 | 1.000 | 0.530 | **0.037** |
| Emotion and Affective Evaluation | -0.373 | 1.000 | 0.338 | 0.217 |
| Speech | 0.612 | **0.037** | -0.706 | 1.000 |
| Reward | -0.641 | 1.000 | 0.508 | **0.041** |
| Emotion Regulation | -0.171 | 1.000 | 0.034 | 0.887 |
| Cognitive task demands | 0.596 | **0.037** | -0.647 | 1.000 |
| Episodic Memory | -0.246 | 1.000 | 0.297 | 0.230 |
| Working_memory | 0.363 | 0.217 | -0.273 | 1.000 |
| Learning and training | 0.234 | 0.437 | -0.122 | 1.000 |
| Attention | 0.606 | **0.037** | -0.540 | 1.000 |
| Perception | 0.410 | 0.128 | -0.392 | 1.000 |
| Decision making | -0.299 | 1.000 | 0.210 | 0.444 |
| ADHD and Eating Disorders | -0.533 | 1.000 | 0.514 | **0.039** |
| Representation | 0.599 | **0.037** | -0.505 | 1.000 |
| Personality Traits | -0.328 | 1.000 | 0.240 | 0.364 |
| Reasoning | 0.085 | 0.815 | -0.089 | 1.000 |
| Memory Encoding | -0.198 | 1.000 | 0.273 | 0.299 |
| OCD and Dopamine Treatment | -0.555 | 1.000 | 0.452 | 0.070 |
| Substance Abuse and Stress Response | -0.610 | 1.000 | 0.570 | **0.037** |
| Recognition Memory | -0.022 | 1.000 | 0.153 | 0.599 |
| Response inhibition | -0.017 | 1.000 | -0.012 | 1.000 |
| Social Cognition and Empathy | 0.057 | 0.887 | -0.139 | 1.000 |

**Notes**: Statistical significance was determined using one-sided spatial permutation test (1,000 times), with Benjamini-Hochberg false-discovery-rate (FDR) correction applied.

**Supplementary Table 8. Statistics for the relationship between baseline tract-based BAGs and school grade at 2-y-follow-up.**

| Tract-based BAG | F | $\beta$ [95% CI] | $\Delta R^2_{adjusted}$ | $p_{FDR}$ |
|---|---|---|---|---|
| Whole-brain | 23.760 | -0.070 [-0.099, -0.042] | 0.286 | < 0.001 |
| AF | 41.194 | -0.054 [-0.071, -0.038] | 0.503 | < 0.001 |
| FAT | 31.234 | -0.044 [-0.060, -0.029] | 0.379 | < 0.001 |
| PAT | 52.488 | -0.040 [-0.051, -0.029] | 0.644 | < 0.001 |
| SLF2 | 44.426 | -0.036 [-0.047, -0.025] | 0.544 | < 0.001 |
| SLF3 | 33.153 | -0.033 [-0.045, -0.022] | 0.403 | < 0.001 |
| SLF1 | 37.310 | -0.059 [-0.078, -0.040] | 0.455 | < 0.001 |
| IFOF | 7.415 | -0.030 [-0.051, -0.008] | 0.081 | 0.006 |
| ILF | 16.250 | -0.035 [-0.051, - 0.018] | 0.192 | < 0.001 |
| UF | 5.560 | -0.019 [-0.035, -0.003] | 0.057 | 0.018 |
| MdLF | 41.015 | -0.062 [-0.080, -0.043] | 0.501 | < 0.001 |
| VOF | 8.536 | -0.019 [-0.031, -0.006] | 0.095 | 0.003 |
| OR | 11.883 | -0.043 [-0.067, -0.019] | 0.137 | 0.001 |
| CST | 17.278 | -0.046 [-0.067, -0.024] | 0.205 | < 0.001 |
| C_PHP | 10.268 | -0.039 [-0.063, -0.015] | 0.117 | 0.001 |
| C_PH | 5.628 | -0.029 [-0.053, -0.005] | 0.058 | 0.018 |
| C_FP | 26.583 | -0.061 [-0.084, -0.038] | 0.321 | < 0.001 |
| C_FPH | 0.001 | 0.000 [-0.021, 0.020] | -0.013 | 0.974 |
| C_PO | 1.614 | -0.016 [-0.039, 0.008] | 0.008 | 0.204 |
| F | 25.278 | -0.065 [-0.090, -0.040] | 0.305 | < 0.001 |
| CS_A | 0.724 | -0.004 [-0.014, 0.006] | -0.003 | 0.395 |
| CS_P | 13.466 | -0.039 [-0.060, -0.018] | 0.157 | < 0.001 |
| CS_S | 8.158 | -0.032 [-0.053, -0.010] | 0.090 | 0.004 |
| TR_A | 20.614 | -0.046 [-0.066, -0.026] | 0.246 | < 0.001 |
| TR_P | 22.980 | -0.046 [-0.065, -0.027] | 0.276 | < 0.001 |
| TR_S | 2.758 | -0.011 [-0.024, 0.002] | 0.022 | 0.097 |
| CC_Body | 8.981 | -0.024 [-0.040, -0.008] | 0.100 | 0.003 |
| CC_Major | 8.817 | -0.021 [-0.035, -0.007] | 0.098 | 0.003 |
| CC_Minor | 17.030 | -0.047 [-0.069, -0.025] | 0.201 | < 0.001 |
| CC_Tap | 4.154 | -0.027 [-0.053, -0.001] | 0.040 | 0.042 |

**Notes:** Statistical significance was determined using two-sided generalized additive model test, with Benjamini-Hochberg false-discovery-rate (FDR) correction applied. Exact p values with FDR correction were provided in Source Data File.

**Supplementary Table 9. Statistics for the relationship between baseline tract-based BAGs and math ability (assessed via Stanford Mental Arithmetic Response Time Evaluation [SAMRTE]) at 3-y-follow-up.**

| Tract-based BAG | F | $\beta$ [95% CI] | $\Delta R^2_{adjusted}$ | $p_{FDR}$ |
|---|---|---|---|---|
| Whole-brain | 64.320 | 1.519 [1.148, 1.890] | 1.063 | < 0.001 |
| AF | 49.573 | 0.792 [0.572, 1.013] | 0.817 | < 0.001 |
| FAT | 68.074 | 0.863 [0.658, 1.068] | 1.125 | < 0.001 |
| PAT | 53.814 | 0.542 [0.397, 0.687] | 0.888 | < 0.001 |
| SLF2 | 47.380 | 0.495 [0.354, 0.636] | 0.781 | < 0.001 |
| SLF3 | 44.205 | 0.512 [0.361, 0.663] | 0.728 | < 0.001 |
| SLF1 | 35.552 | 0.759 [0.509, 1.008] | 0.583 | < 0.001 |
| IFOF | 46.138 | 0.966 [0.687, 1.244] | 0.760 | < 0.001 |
| ILF | 54.206 | 0.828 [0.608, 1.049] | 0.895 | < 0.001 |
| UF | 19.340 | 0.471 [0.261, 0.682] | 0.310 | < 0.001 |
| MdLF | 36.188 | 0.766 [0.516, 1.015] | 0.594 | < 0.001 |
| VOF | 5.809 | 0.203 [0.038, 0.368] | 0.082 | 0.017 |
| OR | 35.647 | 0.981 [0.659, 1.303] | 0.585 | < 0.001 |
| CST | 32.009 | 0.819 [0.536, 1.103] | 0.523 | < 0.001 |
| C_PHP | 34.129 | 0.938 [0.623, 1.253] | 0.559 | < 0.001 |
| C_PH | 16.803 | 0.669 [0.349, 0.989] | 0.267 | < 0.001 |
| C_FP | 23.020 | 0.757 [0.448, 1.066] | 0.372 | < 0.001 |
| C_FPH | 1.832 | 0.184 [-0.083, 0.451] | 0.014 | 0.176 |
| C_PO | 6.847 | 0.423 [0.106, 0.740] | 0.099 | 0.010 |
| F | 32.605 | 0.971 [0.638, 1.305] | 0.533 | < 0.001 |
| CS_A | 15.221 | 0.262 [0.131, 0.394] | 0.241 | < 0.001 |
| CS_P | 89.388 | 1.310 [1.039, 1.582] | 1.477 | < 0.001 |
| CS_S | 32.744 | 0.833 [0.547, 1.118] | 0.536 | < 0.001 |
| TR_A | 48.453 | 0.922 [0.663, 1.182] | 0.799 | < 0.001 |
| TR_P | 44.023 | 0.843 [0.594, 1.092] | 0.725 | < 0.001 |
| TR_S | 6.364 | 0.214 [0.048, 0.380] | 0.091 | < 0.001 |
| CC_Body | 28.155 | 0.566 [0.357, 0.775] | 0.459 | < 0.001 |
| CC_Major | 28.701 | 0.506 [0.321, 0.691] | 0.468 | < 0.001 |
| CC_Minor | 19.521 | 0.669 [0.372, 0.966] | 0.313 | < 0.001 |
| CC_Tap | 27.416 | 0.927 [0.580, 1.274] | 0.446 | < 0.001 |

**Notes**: Statistical significance was determined using two-sided generalized additive model test, with Benjamini-Hochberg false-discovery-rate (FDR) correction applied. Exact p values with FDR correction were provided in Source Data File.

**Supplementary Table 10. Statistics for the relationship between baseline tract-based BAGs and overall accuracy of emotional Stroop task at 3-y-follow-up**

| Tract-based BAG | F | $\beta$ [95% CI] | $\Delta R^2_{adjusted}$ | $p_{FDR}$ |
|---|---|---|---|---|
| Whole-brain | 26.364 | 0.067 [0.041, 0.092] | 0.418 | < 0.001 |
| AF | 16.615 | 0.019 [0.010, 0.029] | 0.258 | < 0.001 |
| FAT | 38.334 | 0.027 [0.019, 0.036] | 0.614 | < 0.001 |
| PAT | 22.028 | 0.015 [0.009, 0.021] | 0.347 | < 0.001 |
| SLF2 | 23.496 | 0.015 [0.009, 0.021] | 0.371 | < 0.001 |
| SLF3 | 31.577 | 0.018 [0.012, 0.025] | 0.504 | < 0.001 |
| SLF1 | 11.657 | 0.018 [0.008, 0.029] | 0.176 | 0.002 |
| IFOF | 5.801 | 0.015 [0.003, 0.026] | 0.079 | 0.027 |
| ILF | 14.952 | 0.018 [0.009, 0.028] | 0.230 | < 0.001 |
| UF | 4.973 | 0.010 [0.001, 0.019] | 0.066 | 0.039 |
| MdLF | 15.459 | 0.021 [0.011, 0.032] | 0.239 | < 0.001 |
| VOF | 1.895 | 0.005 [-0.002, 0.012] | 0.015 | 0.195 |
| OR | 0.208 | 0.003 [-0.010, 0.017] | -0.013 | 0.670 |
| CST | 11.263 | 0.021 [0.009, 0.032] | 0.170 | 0.002 |
| C_PHP | 5.597 | 0.016 [0.003, 0.029] | 0.076 | 0.028 |
| C_PH | 7.269 | 0.019 [0.005, 0.032] | 0.104 | 0.015 |
| C_FP | 3.403 | 0.012 [-0.001, 0.025] | 0.040 | 0.086 |
| C_FPH | 0.024 | 0.001 [-0.010, 0.012] | -0.016 | 0.876 |
| C_PO | 6.805 | 0.018 [0.004, 0.031] | 0.096 | 0.018 |
| F | 6.307 | 0.018 [0.004, 0.032] | 0.088 | 0.023 |
| CS_A | 1.092 | 0.003 [-0.003, 0.009] | 0.002 | 0.329 |
| CS_P | 19.941 | 0.026 [0.015, 0.038] | 0.313 | < 0.001 |
| CS_S | 3.188 | 0.011 [-0.001, 0.023] | 0.036 | 0.093 |
| TR_A | 8.380 | 0.016 [0.005, 0.027] | 0.122 | 0.009 |
| TR_P | 15.838 | 0.021 [0.011, 0.032] | 0.245 | < 0.001 |
| TR_S | 0.650 | -0.003 [-0.010, 0.004] | -0.006 | 0.450 |
| CC_Body | 5.771 | 0.011 [0.002, 0.020] | 0.079 | 0.027 |
| CC_Major | 4.718 | 0.009 [0.001, 0.017] | 0.062 | 0.043 |
| CC_Minor | 3.375 | 0.012 [-0.001, 0.024] | 0.039 | 0.086 |
| CC_Tap | 2.290 | 0.011 [-0.003, 0.026] | 0.021 | 0.156 |

**Notes**: Statistical significance was determined using two-sided generalized additive model test, with Benjamini-Hochberg false-discovery-rate (FDR) correction applied. Exact p values with FDR correction were provided in Source Data File.

**Supplementary Table 11**. Generalized linear model (GLM) analysis results on the relationships between baseline tract-BAG measures (with age-bias correction and including age as the covariate) and the cumulative number of KSADS-5 at baseline, at 2-y-follow-up, and the transition of the transdiagnostic status between baseline and 2-y-follow-up.

| Tract-based measure | $BAG_{Baseline} \sim KSADS_{baseline}$ | | $BAG_{Baseline} \sim KSADS_{followup}$ | | $BAG_{Baseline} \sim KSADS_{conversion}$ | |
|---|---|---|---|---|---|---|
| | F | $p_{FDR}$ | F | $p_{FDR}$ | F | $p_{FDR}$ |
| Whole-brain | 7.05 | 0.004 | 7.03 | 0.005 | 6.06 | 0.002 |
| AF | 9.11 | 0.001 | 4.29 | 0.038 | 7.04 | 0.001 |
| FAT | 9.78 | 0.001 | 8.64 | 0.002 | 7.27 | 0.001 |
| PAT | 13.89 | $1.52 \times 10^{-5}$ | 8.44 | 0.002 | 8.58 | $1.76 \times 10^{-4}$ |
| SLF2 | 8.21 | 0.002 | 5.24 | 0.020 | 4.30 | 0.014 |
| SLF3 | 5.13 | 0.022 | 2.15 | 0.175 | 4.43 | 0.014 |
| SLF1 | 7.49 | 0.003 | 8.75 | 0.002 | 8.04 | $2.57 \times 10^{-4}$ |
| IFOF | 5.52 | 0.017 | 3.88 | 0.048 | 2.71 | 0.078 |
| ILF | 3.14 | 0.100 | 1.76 | 0.247 | 0.87 | 0.484 |
| UF | 1.27 | 0.400 | 0.76 | 0.503 | 0.88 | 0.484 |
| MdLF | 3.90 | 0.061 | 2.46 | 0.142 | 4.35 | 0.014 |
| VOF | 1.73 | 0.295 | 0.84 | 0.498 | 1.83 | 0.187 |
| OR | 0.60 | 0.660 | 2.93 | 0.094 | 2.39 | 0.102 |
| CST | 3.69 | 0.064 | 5.32 | 0.020 | 3.11 | 0.054 |
| C_PHP | 0.52 | 0.689 | 2.26 | 0.165 | 1.65 | 0.226 |
| C_PH | 0.93 | 0.514 | 1.05 | 0.436 | 0.95 | 0.473 |
| C_FP | 3.67 | 0.064 | 4.74 | 0.026 | 3.53 | 0.038 |
| C_FPH | 0.33 | 0.770 | 0.64 | 0.545 | 0.32 | 0.811 |
| C_PO | 2.23 | 0.190 | 1.17 | 0.403 | 1.83 | 0.187 |
| F | 1.17 | 0.425 | 4.93 | 0.024 | 4.61 | 0.013 |
| CS_A | 2.27 | 0.190 | 0.33 | 0.718 | 2.46 | 0.097 |
| CS_P | 2.53 | 0.163 | 7.91 | 0.003 | 3.19 | 0.052 |
| CS_S | 0.37 | 0.765 | 0.76 | 0.503 | 1.27 | 0.347 |
| TR_A | 0.73 | 0.603 | 3.49 | 0.061 | 2.04 | 0.154 |
| TR_P | 1.61 | 0.315 | 6.15 | 0.011 | 2.60 | 0.085 |
| TR_S | 0.21 | 0.837 | 0.91 | 0.483 | 1.06 | 0.430 |
| CC_Body | 4.04 | 0.059 | 3.25 | 0.073 | 2.96 | 0.061 |
| CC_Major | 1.30 | 0.400 | 4.08 | 0.043 | 2.92 | 0.061 |
| CC_Minor | 2.51 | 0.163 | 3.57 | 0.060 | 3.48 | 0.038 |
| CC_Tap | 0.00 | 0.997 | 1.58 | 0.281 | 0.39 | 0.786 |

**Notes:** Statistical significance was corrected using Benjamini-Hochberg false-discovery-rate (FDR).

**Supplementary Table 12**. Generalized linear model (GLM) analysis results on the relationships between baseline tract-wise FA measures (with data harmonization) and the cumulative number of KSADS-5 at baseline, at 2-y-follow-up, and the transition of the transdiagnostic status between baseline and 2-y-follow-up.

| | $BAG_{Baseline}$ ~ | | $BAG_{Baseline}$ ~ $KSADS_{followup}$ | | $BAG_{Baseline}$ ~ $KSADS_{conversion}$ | |
|---|---|---|---|---|---|---|
| | F | $p_{FDR}$ | F | $p_{FDR}$ | F | $p_{FDR}$ |
| Whole-brain | 3.251 | 0.150 | 3.152 | 0.128 | 3.967 | 0.047 |
| AF | 1.508 | 0.369 | 0.464 | 0.786 | 2.145 | 0.137 |
| FAT | 2.001 | 0.254 | 1.945 | 0.268 | 2.900 | 0.090 |
| PAT | 1.308 | 0.386 | 2.797 | 0.166 | 1.162 | 0.387 |
| SLF2 | 4.079 | 0.114 | 1.018 | 0.542 | 2.725 | 0.091 |
| SLF3 | 0.128 | 0.943 | 0.345 | 0.817 | 0.455 | 0.738 |
| SLF1 | 2.345 | 0.206 | 4.449 | 0.070 | 4.313 | 0.036 |
| IFOF | 3.914 | 0.114 | 1.139 | 0.506 | 2.114 | 0.137 |
| ILF | 4.765 | 0.085 | 3.941 | 0.097 | 2.852 | 0.090 |
| UF | 1.091 | 0.420 | 0.560 | 0.769 | 0.777 | 0.563 |
| MdLF | 2.604 | 0.173 | 1.467 | 0.407 | 3.276 | 0.075 |
| VOF | 0.048 | 0.970 | 0.528 | 0.769 | 0.406 | 0.749 |
| OR | 1.375 | 0.383 | 2.612 | 0.169 | 0.960 | 0.474 |
| CST | 5.273 | 0.085 | 12.983 | $7.05 \times 10^{-5}$ | 6.804 | 0.004 |
| C_PHP | 2.900 | 0.150 | 5.137 | 0.044 | 4.617 | 0.036 |
| C_PH | 3.027 | 0.150 | 3.487 | 0.122 | 2.935 | 0.090 |
| C_FP | 2.141 | 0.235 | 1.385 | 0.417 | 2.351 | 0.111 |
| C_FPH | 1.214 | 0.387 | 0.143 | 0.866 | 1.815 | 0.185 |
| C_PO | 1.652 | 0.338 | 0.168 | 0.866 | 2.425 | 0.111 |
| F | 1.234 | 0.387 | 1.982 | 0.268 | 2.779 | 0.091 |
| CS_A | 0.132 | 0.943 | 0.236 | 0.866 | 2.385 | 0.111 |
| CS_P | 5.000 | 0.085 | 3.310 | 0.122 | 3.194 | 0.075 |
| CS_S | 2.989 | 0.150 | 2.696 | 0.169 | 2.352 | 0.111 |
| TR_A | 1.364 | 0.383 | 0.408 | 0.798 | 1.216 | 0.378 |
| TR_P | 0.663 | 0.618 | 2.067 | 0.268 | 2.619 | 0.098 |
| TR_S | 0.556 | 0.662 | 3.366 | 0.122 | 3.343 | 0.075 |
| CC_Body | 3.001 | 0.150 | 5.716 | 0.033 | 4.432 | 0.036 |
| CC_Major | 3.782 | 0.114 | 8.580 | 0.003 | 3.732 | 0.054 |
| CC_Minor | 0.030 | 0.970 | 0.174 | 0.866 | 1.980 | 0.156 |
| CC_Tap | 2.589 | 0.173 | 0.908 | 0.576 | 0.667 | 0.613 |

**Notes:** Statistical significance was corrected using Benjamini-Hochberg false-discovery-rate (FDR).

**Supplementary Table 13**. Sensitivity analysis on the impact of age-bias correction on the relationships between baseline tract-BAG measures (without age-bias correction and including age as the covariate) and the cumulative number of KSADS-5 diagnoses at baseline, at 2-y-follow-up, and the transition of the transdiagnostic status between baseline and 2-y-follow-up.

| Tract-based measure | $BAG_{Baseline}$ ~ $KSADS_{baseline}$ | | $BAG_{Baseline}$ ~ $KSADS_{followup}$ | | $BAG_{Baseline}$ ~ $KSADS_{conversion}$ | |
|---|---|---|---|---|---|---|
| | F | $p_{FDR}$ | F | $p_{FDR}$ | F | $p_{FDR}$ |
| Whole-brain | 6.36 | 0.004 | 6.70 | 0.006 | 5.42 | 0.005 |
| AF | 8.35 | 0.001 | 3.87 | 0.048 | 6.41 | 0.002 |
| FAT | 9.15 | 0.001 | 8.51 | 0.002 | 6.77 | 0.001 |
| PAT | 13.21 | $2.99 \times 10^{-5}$ | 8.30 | 0.002 | 8.07 | $3.69 \times 10^{-4}$ |
| SLF2 | 7.91 | 0.002 | 5.21 | 0.018 | 4.10 | 0.023 |
| SLF3 | 4.79 | 0.019 | 2.00 | 0.205 | 4.15 | 0.023 |
| SLF1 | 6.86 | 0.003 | 8.17 | 0.002 | 7.38 | $6.64 \times 10^{-4}$ |
| IFOF | 4.96 | 0.014 | 3.45 | 0.068 | 2.30 | 0.127 |
| ILF | 2.78 | 0.093 | 1.73 | 0.236 | 0.69 | 0.594 |
| UF | 1.23 | 0.389 | 0.48 | 0.637 | 0.86 | 0.526 |
| MdLF | 3.32 | 0.054 | 2.19 | 0.179 | 3.77 | 0.030 |
| VOF | 1.51 | 0.283 | 0.80 | 0.525 | 1.62 | 0.253 |
| OR | 0.38 | 0.652 | 2.49 | 0.139 | 1.95 | 0.182 |
| CST | 3.21 | 0.058 | 5.36 | 0.017 | 2.80 | 0.083 |
| C_PHP | 0.29 | 0.682 | 1.80 | 0.230 | 1.23 | 0.366 |
| C_PH | 0.93 | 0.505 | 0.84 | 0.525 | 0.72 | 0.594 |
| C_FP | 3.09 | 0.058 | 4.52 | 0.032 | 2.96 | 0.083 |
| C_FPH | 0.20 | 0.767 | 0.67 | 0.563 | 0.20 | 0.893 |
| C_PO | 1.65 | 0.181 | 0.85 | 0.525 | 1.28 | 0.358 |
| F | 0.73 | 0.416 | 4.38 | 0.033 | 3.83 | 0.030 |
| CS_A | 2.07 | 0.181 | 0.27 | 0.760 | 2.30 | 0.127 |
| CS_P | 2.21 | 0.153 | 7.78 | 0.003 | 2.84 | 0.083 |
| CS_S | 0.53 | 0.759 | 0.61 | 0.577 | 1.28 | 0.358 |
| TR_A | 0.59 | 0.594 | 3.29 | 0.075 | 1.81 | 0.208 |
| TR_P | 1.33 | 0.304 | 6.13 | 0.009 | 2.24 | 0.130 |
| TR_S | 0.21 | 0.835 | 0.78 | 0.525 | 0.98 | 0.476 |
| CC_Body | 3.74 | 0.051 | 3.14 | 0.082 | 2.74 | 0.083 |
| CC_Major | 1.16 | 0.389 | 3.88 | 0.048 | 2.74 | 0.083 |
| CC_Minor | 1.89 | 0.153 | 2.65 | 0.126 | 2.68 | 0.085 |
| CC_Tap | 0.07 | 0.997 | 1.96 | 0.205 | 0.37 | 0.802 |

**Notes:** Statistical significance was determined using two-sided generalized linear model test, with Benjamini-Hochberg false-discovery-rate (FDR) correction applied.

**Supplementary Table 14**. Sensitivity analysis on the impact of age-bias correction on the relationships between baseline tract-BAG measures (with age-bias correction and without age as the covariate) and the cumulative number of KSADS-5 diagnoses at baseline, at 2-y-follow-up, and the transition of the transdiagnostic status between baseline and 2-y-follow-up.

| Tract-based measure | $BAG_{Baseline}$ ~ $KSADS_{baseline}$ | | $BAG_{Baseline}$ ~ $KSADS_{followup}$ | | $BAG_{Baseline}$ ~ $KSADS_{conversion}$ | |
|---|---|---|---|---|---|---|
| | F | $p_{FDR}$ | F | $p_{FDR}$ | F | $p_{FDR}$ |
| Whole-brain | 7.05 | 0.004 | 7.02 | 0.005 | 6.05 | 0.002 |
| AF | 9.11 | 0.001 | 4.29 | 0.038 | 7.04 | 0.001 |
| FAT | 9.78 | $8.54 \times 10^{-4}$ | 8.64 | 0.002 | 7.27 | 0.001 |
| PAT | 13.89 | $2.85 \times 10^{-5}$ | 8.44 | 0.002 | 8.58 | $3.30 \times 10^{-4}$ |
| SLF2 | 8.21 | 0.002 | 5.23 | 0.020 | 4.29 | 0.016 |
| SLF3 | 5.13 | 0.022 | 2.15 | 0.175 | 4.43 | 0.016 |
| SLF1 | 7.48 | 0.003 | 8.74 | 0.002 | 8.03 | $3.65 \times 10^{-4}$ |
| IFOF | 5.51 | 0.017 | 3.87 | 0.048 | 2.71 | 0.082 |
| ILF | 3.13 | 0.101 | 1.75 | 0.247 | 0.87 | 0.486 |
| UF | 1.27 | 0.400 | 0.76 | 0.503 | 0.88 | 0.486 |
| MdLF | 3.89 | 0.061 | 2.46 | 0.142 | 4.35 | 0.016 |
| VOF | 1.73 | 0.295 | 0.84 | 0.498 | 1.83 | 0.191 |
| OR | 0.60 | 0.660 | 2.93 | 0.094 | 2.39 | 0.105 |
| CST | 3.68 | 0.064 | 5.31 | 0.020 | 3.10 | 0.059 |
| C_PHP | 0.52 | 0.689 | 2.26 | 0.165 | 1.65 | 0.230 |
| C_PH | 0.93 | 0.515 | 1.05 | 0.436 | 0.95 | 0.478 |
| C_FP | 3.67 | 0.064 | 4.74 | 0.026 | 3.53 | 0.042 |
| C_FPH | 0.33 | 0.770 | 0.64 | 0.545 | 0.32 | 0.811 |
| C_PO | 2.23 | 0.190 | 1.17 | 0.404 | 1.82 | 0.191 |
| F | 1.16 | 0.427 | 4.91 | 0.025 | 4.59 | 0.016 |
| CS_A | 2.27 | 0.190 | 0.33 | 0.718 | 2.46 | 0.101 |
| CS_P | 2.53 | 0.163 | 7.91 | 0.003 | 3.19 | 0.057 |
| CS_S | 0.37 | 0.765 | 0.76 | 0.503 | 1.27 | 0.352 |
| TR_A | 0.73 | 0.604 | 3.48 | 0.062 | 2.04 | 0.160 |
| TR_P | 1.61 | 0.315 | 6.15 | 0.011 | 2.60 | 0.089 |
| TR_S | 0.21 | 0.838 | 0.91 | 0.484 | 1.06 | 0.437 |
| CC_Body | 4.03 | 0.059 | 3.25 | 0.073 | 2.96 | 0.065 |
| CC_Major | 1.30 | 0.400 | 4.08 | 0.043 | 2.92 | 0.065 |
| CC_Minor | 2.51 | 0.163 | 3.57 | 0.060 | 3.48 | 0.042 |
| CC_Tap | 0.00 | 0.997 | 1.57 | 0.284 | 0.39 | 0.790 |

**Notes:** Statistical significance was determined using two-sided generalized linear model test, with Benjamini-Hochberg false-discovery-rate (FDR) correction applied.

**Supplementary Table 15**. Sensitivity analysis on the impact of including pubertal effects as additional covariate on the relationships between baseline tract-BAG measures (with age-bias correction) and the cumulative number of KSADS-5 diagnoses at baseline, at 2-y-follow-up, and the transition of the transdiagnostic status between baseline and 2-y-follow-up.

| Tract-based measure | $BAG_{Baseline} \sim KSADS_{baseline}$ | | $BAG_{Baseline} \sim KSADS_{followup}$ | | $BAG_{Baseline} \sim KSADS_{conversion}$ | |
|---|---|---|---|---|---|---|
| | F | $p_{FDR}$ | F | $p_{FDR}$ | F | $p_{FDR}$ |
| Association | | | | | | |
| Development Mode | 12.503 | $6.06 \times 10^{-5}$ | 10.754 | 0.001 | 9.380 | $1.11 \times 10^{-4}$ |
| Subcortical/Limbic | | | | | | |
| Development Mode | 4.277 | 0.045 | 5.535 | 0.016 | 3.740 | 0.037 |
| Whole-brain | 5.300 | 0.023 | 5.735 | 0.016 | 5.013 | 0.008 |
| AF | 8.887 | 0.001 | 4.540 | 0.030 | 7.154 | 0.001 |
| FAT | 7.922 | 0.003 | 7.544 | 0.004 | 6.030 | 0.003 |
| PAT | 12.511 | $6.06 \times 10^{-5}$ | 8.110 | 0.003 | 7.918 | $3.05 \times 10^{-4}$ |
| SLF2 | 7.136 | 0.005 | 4.627 | 0.030 | 3.686 | 0.037 |
| SLF3 | 4.620 | 0.040 | 1.994 | 0.198 | 4.077 | 0.027 |
| SLF1 | 7.056 | 0.005 | 8.481 | 0.003 | 8.040 | $3.05 \times 10^{-4}$ |
| IFOF | 4.341 | 0.045 | 3.468 | 0.067 | 2.310 | 0.125 |
| ILF | 2.738 | 0.138 | 1.148 | 0.423 | 0.611 | 0.649 |
| UF | 1.396 | 0.390 | 0.478 | 0.640 | 0.771 | 0.564 |
| MdLF | 2.775 | 0.138 | 2.038 | 0.198 | 3.513 | 0.042 |
| VOF | 1.363 | 0.390 | 0.496 | 0.640 | 1.439 | 0.282 |
| OR | 0.466 | 0.744 | 3.114 | 0.079 | 2.659 | 0.093 |
| CST | 2.995 | 0.134 | 5.074 | 0.022 | 2.691 | 0.093 |
| C_PHP | 0.405 | 0.762 | 2.022 | 0.198 | 1.524 | 0.273 |
| C_PH | 1.138 | 0.466 | 0.577 | 0.619 | 0.769 | 0.564 |
| C_FP | 2.792 | 0.138 | 4.361 | 0.032 | 2.956 | 0.077 |
| C_FPH | 0.109 | 0.897 | 0.607 | 0.619 | 0.191 | 0.902 |
| C_PO | 1.648 | 0.356 | 0.681 | 0.600 | 1.620 | 0.254 |
| F | 0.588 | 0.684 | 4.497 | 0.030 | 5.182 | 0.008 |
| CS_A | 1.510 | 0.372 | 0.058 | 0.943 | 2.250 | 0.129 |
| CS_P | 1.608 | 0.356 | 7.333 | 0.004 | 2.733 | 0.093 |
| CS_S | 0.684 | 0.646 | 0.712 | 0.600 | 1.497 | 0.273 |
| TR_A | 0.183 | 0.889 | 3.150 | 0.079 | 1.874 | 0.192 |
| TR_P | 1.071 | 0.477 | 5.560 | 0.016 | 2.169 | 0.136 |
| TR_S | 0.131 | 0.897 | 1.446 | 0.328 | 1.254 | 0.342 |
| CC_Body | 3.636 | 0.077 | 2.804 | 0.102 | 2.613 | 0.093 |
| CC_Major | 0.854 | 0.568 | 3.623 | 0.061 | 2.569 | 0.093 |
| CC_Minor | 1.705 | 0.356 | 3.357 | 0.070 | 3.111 | 0.067 |
| CC_Tap | 0.217 | 0.888 | 1.023 | 0.460 | 0.545 | 0.673 |

**Notes:** Statistical significance was determined using two-sided generalized linear model test, with Benjamini-Hochberg false-discovery-rate (FDR) correction applied.

**Supplementary Table 16**. Sensitivity analysis on the impact of including socioeconomic status (SES) as additional covariate on the relationships between baseline tract-BAG measures (with age-bias correction) and the cumulative number of KSADS-5 diagnoses at baseline, at 2-y-follow-up, and the transition of the transdiagnostic status between baseline and 2-y-follow-up.

| Tract-based measure | $BAG_{Baseline} \sim KSADS_{baseline}$ | | $BAG_{Baseline} \sim KSADS_{followup}$ | | $BAG_{Baseline} \sim KSADS_{conversion}$ | |
|---|---|---|---|---|---|---|
| | F | $p_{FDR}$ | F | $p_{FDR}$ | F | $p_{FDR}$ |
| Association Development Mode | 15.216 | $8.10 \times 10^{-6}$ | 11.817 | $2.40 \times 10^{-4}$ | 10.641 | $1.79 \times 10^{-5}$ |
| Subcortical/Limbic Development Mode | 6.238 | 0.008 | 6.986 | 0.005 | 4.770 | 0.012 |
| Whole-brain | 7.083 | 0.004 | 6.902 | 0.005 | 6.086 | 0.002 |
| AF | 9.180 | $8.32 \times 10^{-4}$ | 4.193 | 0.037 | 7.096 | 0.001 |
| FAT | 9.844 | $5.73 \times 10^{-4}$ | 8.669 | 0.002 | 7.323 | 0.001 |
| PAT | 14.004 | $1.36 \times 10^{-5}$ | 8.410 | 0.002 | 8.657 | $1.58 \times 10^{-4}$ |
| SLF2 | 8.252 | 0.002 | 5.305 | 0.016 | 4.316 | 0.014 |
| SLF3 | 5.160 | 0.018 | 2.173 | 0.166 | 4.462 | 0.014 |
| SLF1 | 7.523 | 0.003 | 8.574 | 0.002 | 8.073 | $2.44 \times 10^{-4}$ |
| IFOF | 5.533 | 0.014 | 3.742 | 0.051 | 2.716 | 0.077 |
| ILF | 3.143 | 0.092 | 1.734 | 0.246 | 0.877 | 0.482 |
| UF | 1.279 | 0.387 | 0.651 | 0.556 | 0.886 | 0.482 |
| MdLF | 3.911 | 0.053 | 2.415 | 0.143 | 4.367 | 0.014 |
| VOF | 1.732 | 0.283 | 0.822 | 0.502 | 1.828 | 0.187 |
| OR | 0.600 | 0.650 | 2.858 | 0.097 | 2.400 | 0.100 |
| CST | 3.688 | 0.058 | 5.356 | 0.016 | 3.107 | 0.054 |
| C_PHP | 0.518 | 0.681 | 2.184 | 0.166 | 1.653 | 0.224 |
| C_PH | 0.932 | 0.504 | 0.958 | 0.461 | 0.955 | 0.472 |
| C_FP | 3.683 | 0.058 | 4.730 | 0.025 | 3.539 | 0.037 |
| C_FPH | 0.330 | 0.767 | 0.618 | 0.556 | 0.320 | 0.811 |
| C_PO | 2.231 | 0.181 | 1.014 | 0.461 | 1.826 | 0.187 |
| F | 1.170 | 0.414 | 4.676 | 0.025 | 4.633 | 0.012 |
| CS_A | 2.275 | 0.181 | 0.278 | 0.757 | 2.469 | 0.096 |
| CS_P | 2.541 | 0.153 | 7.842 | 0.003 | 3.204 | 0.051 |
| CS_S | 0.374 | 0.759 | 0.719 | 0.538 | 1.274 | 0.346 |
| TR_A | 0.733 | 0.591 | 3.361 | 0.065 | 2.050 | 0.152 |
| TR_P | 1.614 | 0.304 | 6.103 | 0.009 | 2.600 | 0.085 |
| TR_S | 0.212 | 0.835 | 0.943 | 0.461 | 1.064 | 0.430 |
| CC_Body | 4.036 | 0.052 | 3.216 | 0.071 | 2.963 | 0.061 |
| CC_Major | 1.297 | 0.387 | 4.026 | 0.041 | 2.922 | 0.061 |
| CC_Minor | 2.511 | 0.153 | 3.541 | 0.058 | 3.480 | 0.037 |
| CC_Tap | 0.003 | 0.997 | 1.615 | 0.265 | 0.389 | 0.786 |

**Notes:** Statistical significance was determined using two-sided generalized linear model test, with Benjamini-Hochberg false-discovery-rate (FDR) correction applied.

**Supplementary Table 17. Scanning parameters of T1w images in HCP-D, ABCD and HBN datasets.**

**(a)**

| | HCP-D | ABCD | | |
|---|---|---|---|---|
| Scanner | Siemens Prisma | Siemens Prisma | GE 750 | Philips Achieva |
| Sequence | Multi-echo T1w MPRAGE | MPRAGE | | |
| TR/TE (ms) | 2500/(1.8/3.6/5.4/7.2) | 2500/2.88 | 2500/2 | 6.31/2.9 |
| TI (ms) | 1000 | 1060 | 1060 | 1060 |
| Flip angle (°) | 8 | 8 | 8 | 8 |
| FOV | 256 × 240 × 166 | 256 × 256 | 256 × 256 | 256 × 240 |
| Resolution (mm) | 0.8 × 0.8 × 0.8 | 1.0 × 1.0 × 1.0 | 1.0 × 1.0 × 1.0 | 1.0 × 1.0 × 1.0 |

**(b)**

| Site | RUBIC | CBIC | CCNY |
|---|---|---|---|
| Scanner | Siemens Tim Trio | Siemens Prisma | Siemens Prisma |
| Sequence | | MPRAGE | |
| TR/TE (ms) | | 2500/3.15 | |
| TI (ms) | | 1060 | |
| Flip angle (°) | | 8 | |
| %FOV Phase | | 100% | |
| Resolution (mm) | | 0.8 × 0.8 × 0.8 | |

Abbreviation: RUBIC: Rutgers University Brain Imaging Center; CBIC: CitiGroup Cornell Brain Imaging Center; CUNY: City College of New York

**Supplementary Table 18. Scanning parameters of dMRI images in HCP-D, ABCD and HBN datasets.**

**(a)**

| Dataset | HCP-D | ABCD | | |
|---|---|---|---|---|
| Scanner | Siemens 3T Prisma | Siemens 3T Prisma | GE 3T 750 | Philips 3T Achieva |
| Sequence | Multiband EPI | | HARDI | |
| TR/TE (ms) | 3230/89.20 | 4100/88 | 4100/81.9 | 5300/89 |
| Flip angle (°) | 78 | 90 | 78 | 77 |
| b-values ($s/mm^2$) | 1500 (186-dirs)<br>3000 (184-dirs) | | 500 (6-dirs)<br>1000 (15-dirs)<br>2000 (15-dirs)<br>3000 (60-dirs) | |
| FOV | 210 × 210 | 240 × 240 | 240 × 240 | 240 × 240 |
| Resolution (mm) | 1.5 × 1.5 × 1.5 | 1.7 × 1.7 × 1.7 | 1.7 × 1.7 × 1.7 | 1.7 × 1.7 × 1.7 |
| Multiband acceleration | 4 | 3 | 3 | 3 |

**(b)**

| Site | RUBIC | CBIC | CCNY |
|---|---|---|---|
| Scanner | Siemens Tim Trio | Siemens Prisma | Siemens Prisma |
| TR/TE (ms) | | 3320/100.2 | |
| Flip angle (°) | | 90 | |
| b-values ($s/mm^2$) | | 1000 (64-dirs)<br>2000 (64-dirs) | |
| %FOV phase | | 100% | |
| Resolution (mm) | | 1.8 × 1.8 × 1.8 | |
| Multiband acceleration | | 3 | |

Abbreviation: RUBIC: Rutgers University Brain Imaging Center; CBIC: CitiGroup Cornell Brain Imaging Center; CUNY: City College of New York

**Supplementary Table 19**. **Neurocognitive measures and the corresponding abbreviations in this study**

| Test | Subscale (measure) | Abbreviation | ABCD field |
|---|---|---|---|
| NIH Toolbox cognition battery[2,3] | Picture Vocabulary Test Score | nihtbx_pic_vocabulary | nihtbx_picvocab_uncorrected |
| | Flanker Test Score | nihtbx_flanker | nihtbx_flanker_uncorrected |
| | Dimensional Change Card Sort Test Score | nihtbx_card_sort | nihtbx_cardsort_uncorrected |
| | List Sorting Working Memory | nihtbx_list_sort | nihtbx_list_uncorrected |
| | Oral Reading Recognition Test Score | nihtbx_oral_reading | nihtbx_reading_uncorrected |
| | Pattern Comparison Processing Speed | nihtbx_pattern_comp | nihtbx_pattern_uncorrected |
| | Picture Sequence Memory Test Score | nihtbx_pic_seq_memory | nihtbx_picvocab_uncorrected |
| | Fluid Intelligence | nihtbx_fluidcomp | nihtbx_fluidcomp_uncorrected |
| | Crystal Intelligence | nihtbx_cryst_comp | nihtbx_cryst_uncorrected |
| | Total Intelligence | nihtbx_total_comp | nihtbx_totalcomp_uncorrected |
| Rey Auditory Verbal Learning Test (RAVLT) | Accuracy in the immediate test | verbal_learning_immediate | pea_ravlt_sd_trial_vi_tc |
| | Accuracy in the delayed test | verbal_learning_delayed | pea_ravlt_ld_trial_vii_tc |
| Cash choice task | Choice of short Smaller-sooner | cash_choice_prefer_ss | cash_choice_prefer_ss |
| | Choice of short Larger-later | cash_choice_prefer_ll | cash_choice_prefer_ll |
| Wechsler Intelligence Scale for Children-V (WISC-V) | Matrix Reasoning Test Scaled Score | matrix_reasoning | pea_wiscv_tss |
| Little Man Task | Efficiency ratio | little_man_efficiency | lmt_scr_efficiency |
| Recognition memory test | d’ | recog_memory | |
| fMRI tasks | Emotional n-back (% accuracy on 2-back - % accuracy on 0-back) | 2_back | tfmri_nb_all_beh_c2b_rate |
| | Stop Signal Reaction Time, mean estimation | SST_ssrt | tfmri_sst_all_beh_total_mssrt |
| | Monetary Incentive Delay (Mean earnings) | MID_total_earning | tfmri_mid_all_beh_t_earnings |

**Supplementary Table 20**. **Psychopathology-related measures and the corresponding abbreviations in this study**

| Questionnaire | Subscale (measure) | Abbreviation | ABCD field (ABCD file) |
|---|---|---|---|
| Prodromal Questionnaire Brief Version (PQ-B)[4] | Severity score of prodromal psychosis | PQB_psychosis_severity | pps_y_ss_severity_score |
| Ten-item Mania Scale[5] | Total score of mania | PGBI_mania_total | pgbi_p_ss_score |
| Children Behavior Check List (CBCL)[6] | Anxious/Depressive symptoms | CBCL_syn_anxdep | cbcl_scr_syn_anxdep_r |
| | Withdrawn/Depressed symptoms | CBCL_syn_withdep | cbcl_scr_syn_withdep_r |
| | Somatic Complaints | CBCL_syn_somatic | cbcl_scr_dsm5_somaticpr_r |
| | Social Problems | CBCL_syn_social_prob | cbcl_scr_syn_social_r |
| | Thought Problems | CBCL_syn_thought_prob | cbcl_scr_syn_thought_r |
| | Attention Problems | CBCL_syn_attention_prob | cbcl_scr_syn_attention_r |
| | Rule-Breaking Behavior | CBCL_syn_rulebreak | cbcl_scr_syn_rulebreak_r |
| | Aggressive Behavior | CBCL_syn_aggresive | cbcl_scr_syn_aggressive_r |
| | Internalizing Problems | CBCL_internal | cbcl_scr_syn_internal_r |
| | Externalizing Problems | CBCL_external | cbcl_scr_syn_external_r |
| | Total Problems | CBCL_total | cbcl_scr_syn_totprob_r |
| | Depressive Problems | CBCL_dsm_depressive | cbcl_scr_dsm5_depress_r |
| | Anxiety Problems | CBCL_dsm_anxiety | cbcl_scr_dsm5_anxdisord_r |
| | Somatic Problems | CBCL_dsm_somatic | cbcl_scr_dsm5_somaticpr_r |
| | Attention-deficit/hyperactivity Disorder | CBCL_dsm_ADHD | cbcl_scr_dsm5_adhd_r |
| | Oppositional Defiant Problems | CBCL_dsm_opposit | cbcl_scr_dsm5_opposit_r |
| | Conduct Problems | CBCL_dsm_conduct_prob | cbcl_scr_dsm5_conduct_r |
| | Sluggish Cognitive Tempo | CBCL_SCT | cbcl_scr_07_sct_r |
| | Obsessive-Compulsive Problems | CBCL_OCD | cbcl_scr_07_ocd_r |
| | Stress Problems | CBCL_stress | cbcl_scr_07_stress_r |
| Urgency, Perseverance, Premeditation and Sensation seeking (UPPS-P)[7,8] | Lack of Planning | UPPS_lack_planing | upps_y_ss_lack_of_planning |
| | Sensation Seeking | UPPS_sensation_seeking | upps_y_ss_sensation_seeking |
| | Positive Urgency | UPPS_positive_urgency | upps_y_ss_positive_urgency |
| | Negative Urgency | UPPS_negative_urgency | upps_y_ss_negative_urgency |
| | Lack of Perseverance | UPPS_lack_perserverance | upps_y_ss_lack_of_perseverance |
| Behavioral Inhibition & Behavioral Activation Scales (BIS/BAS)[9] | Drive | BISBAS_drive | mh_y_bisbas |
| | Reward responsiveness | BISBAS_reward_resp | bis_y_ss_bas_rr |
| | Fun seeking | BISBAS_fun_seek | bis_y_ss_bas_fs |
| | BIS total score | BISBAS_bis_total | bis_y_ss_bis_sum |

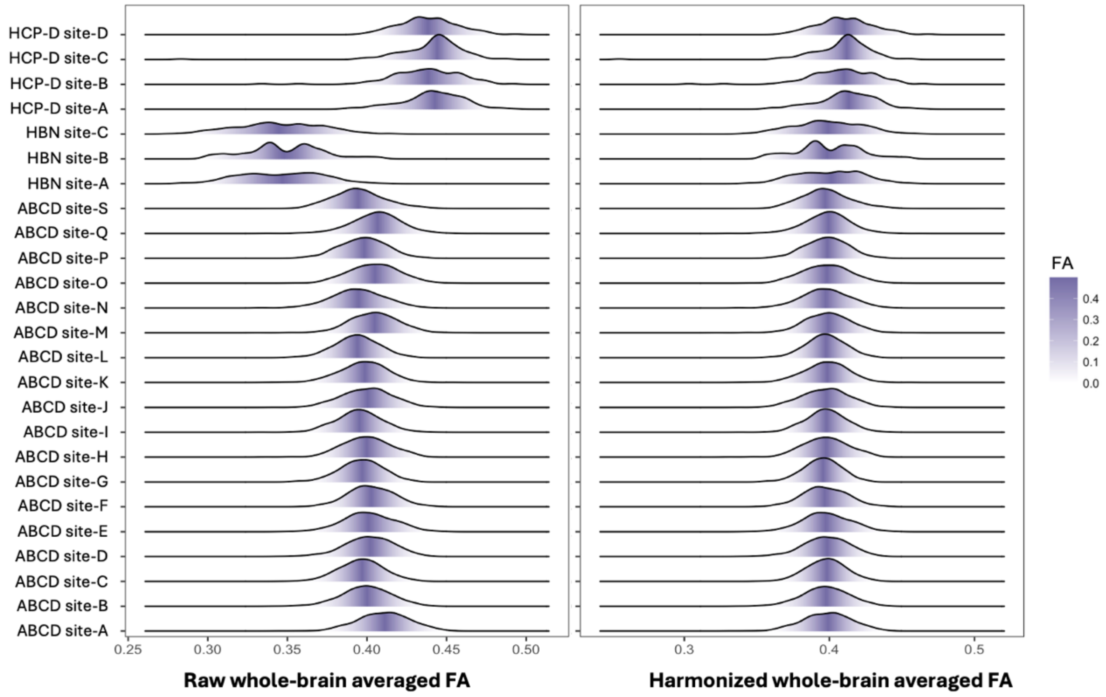


**Supplementary Figure 1. Domain shifts of raw FA values across sites and cohorts and the effects of data harmonization.**

Ridge density plots depict the distributions of raw (left) and harmonized (right) whole-brain averaged FA values across multiple sites in three developmental cohorts (HCP-D, HBN, and ABCD). Each row corresponds to a site, with density shading reflecting the relative frequency of FA values (darker = higher density). Prior to harmonization, noticeable site-related shifts and variability in FA distributions were evident. After harmonization, distributions became more consistent across sites. Abbreviations: FA: fractional anisotropy. HCP-D, Human Connectome Project in Development); ABCD, Adolescent Brain Cognitive Development; HBN, Healthy Brain Network pediatric mental health.

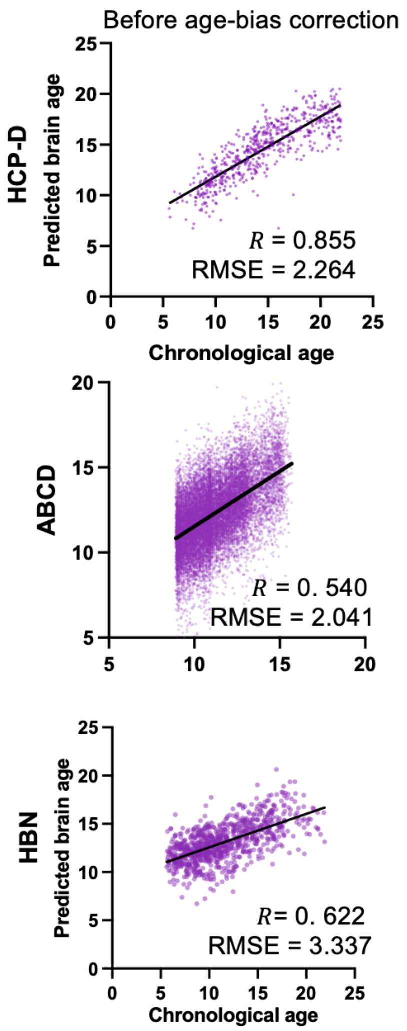


**Supplementary Figure 2. Associations between the age predicted by whole-brain tracts and chronological age in cross-validation and independent testing.**

Scatter plots show the relationships between predicted brain age and chronological age using whole-brain tract-profiles from the Human Connectome Project in Development (HCP-D), Adolescent Brain Cognitive Development (ABCD) and Healthy Brain Network (HBN) pediatric mental health studies, respectively.

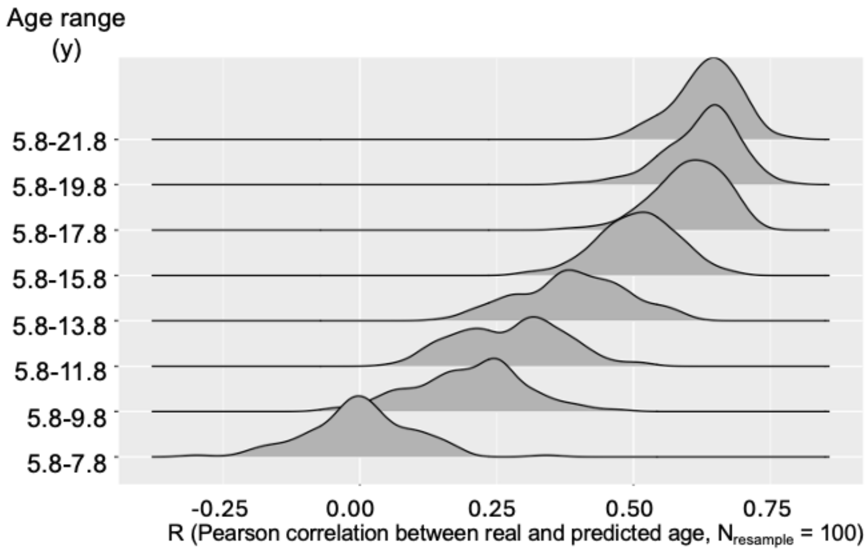


**Supplementary Figure 3. Effect of age range on the predictive performance of brain age model.**

Simulation analyses were conducted using the Healthy Brain Network (HBN) dataset to examine how the age-range of testing samples influences model performance. For each age range (indicated on the y-axis), 100 subjects were randomly sampled for 100 times. The x-axis shows the distributions of Pearson correlations between the predicted ages (whole-brain brain ages without age-bias correction) and chronological ages across iterations.

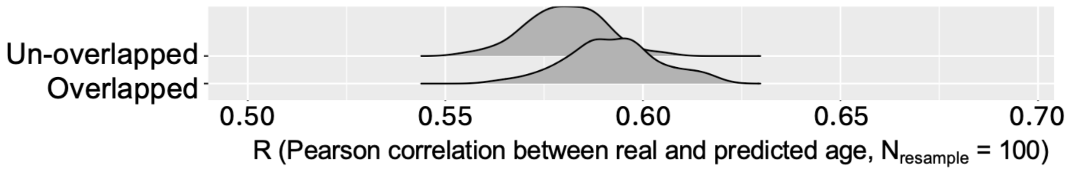


**Supplementary Figure 4. Influence of intra-subject overlap on tract-based brain-age prediction performance.**

Density distributions of prediction accuracy (Pearson's R between chronological and predicted age) across 100 resampling iterations of ABCD participants. Each iteration sampled 500 participants per timepoint using either (i) non-overlapping samples (each subject included once across all time points) or (ii) overlapping samples (subjects allowed to appear in all time points). The two sampling strategies yielded highly similar predictive performance, with mean R values of 0.580 ± 0.016 for non-overlapping samples and 0.592 ± 0.010 for overlapping samples

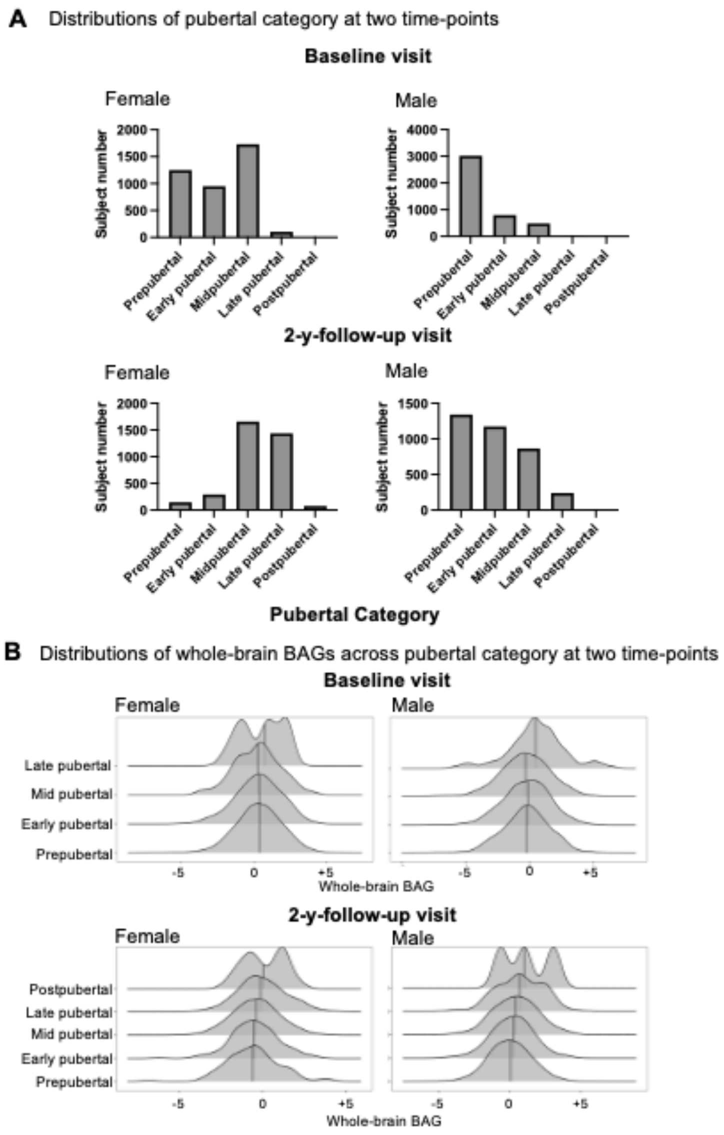


**Supplementary Figure 5**. **Pubertal stage distributions and their associations with whole-brain BAGs.**

(A) Distributions of pubertal categories (prepubertal, early pubertal, mid pubertal, late pubertal, and postpubertal) at baseline and 2-year follow-up visits in females and males. Subject counts within each category are shown separately by sex and timepoint.

(B) Distributions of whole-brain brain age gaps (BAGs) across pubertal categories at baseline (top) and 2-year follow-up (bottom), stratified by sex. Ridge plots illustrate how BAGs vary across pubertal stages, with earlier categories (prepubertal/early pubertal) generally showing younger predicted brain age relative to chronological age compared to later stages.

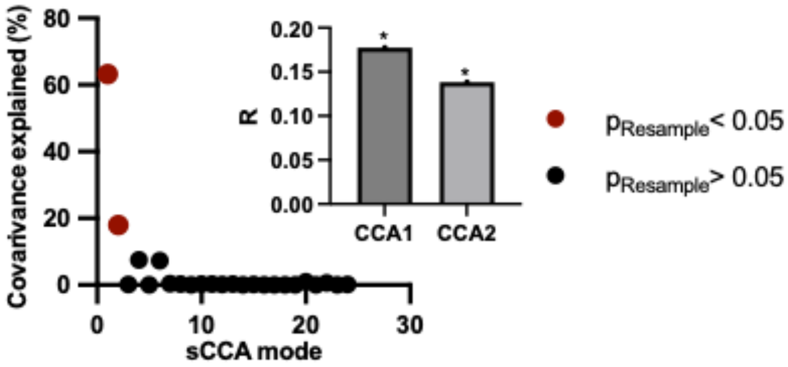


**Supplementary Figure 6. Covariance explained by the canonical variates.**
The first two canonical variates were statistically significant, as determined by permutation testing with false discovery rate (FDR) correction (two-tailed $p < 0.05$). Source data are provided as a Source Data file.

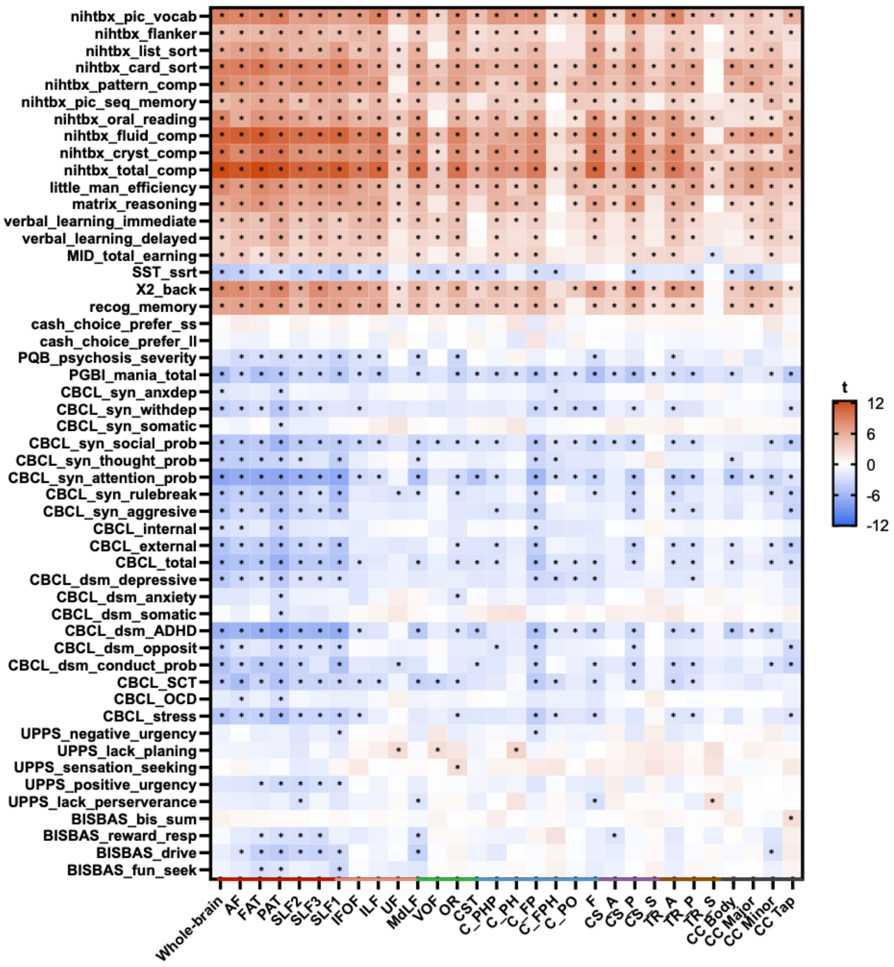


**Supplementary Figure 7**. **Heatmap of univariate associations between tract-based BAGs (with age-bias correction) and behavioral measures**.

The t-values from the Generalized Linear Model (GLM) analysis are shown. Asterisks indicate two-tailed $p < 0.05$ after False Discovery Rate (FDR) correction. Abbreviation: BAG, Brain Age Gap; AF, Arcuate Fasciculus; FAT, Frontal Aslant Tract; PAT, Parietal Aslant Tract; SLF, Superior Longitudinal Fasciculus; IFOF, Inferior Fronto Occipital Fasciculus; ILF, Inferior Longitudinal Fasciculus; UF, Uncinate Fasciculus; MdLF, Middle Longitudinal Fasciculus; C_FPH, Cingulum Frontal Parahippocampal; C_FP, Cingulum Frontal Parietal; C_PH, Cingulum Parahippocampal; C_PHP, Cingulum Parahippocampal Parietal; C_PO, Cingulum Parolfactory; F, Fornix; CST, Corticospinal Tract; OR, Optic Radiation; VOF, Vertical Occipital Fasciculus; TR_A, Thalamic Radiation Anterior; TR_P, Thalamic Radiation Posterior; TR_S, Thalamic Radiation Superior; CS_A, Corticostriatal Tract Anterior; CS_P, Corticostriatal Tract Posterior; CS_S, Corticostriatal Tract Superior; CC, Corpus Callosum; CC_Body, CC Body; CC_Major, CC Forceps Major; CC_Minor, CC Forceps Minor; CC_Tap, CC Tapetum; nihtbx: NIH Toolbox; MID: Monetary Incentive Delay; SST_ssrt: Stop Signal Reaction Time of Stop Signal Task; PQB: Prodromal Questionnaire Brief Version; PGBI: Parent General Behavior Inventory; CBCL: Children Behavior Check List; ADHD: Attention-deficit/hyperactivity Disorder; SCT: Sluggish Cognitive Tempo; OCD: Obsessive-compulsive Disorder; UPPS: Urgency, Perseverance, Premeditation and Sensation-seeking; BISBAS: Behavioral Inhibition & Behavioral Activation Scales. See Supplementary Table 19 and 20 for more details of the behavioral measures. Source data are provided as a Source Data file.

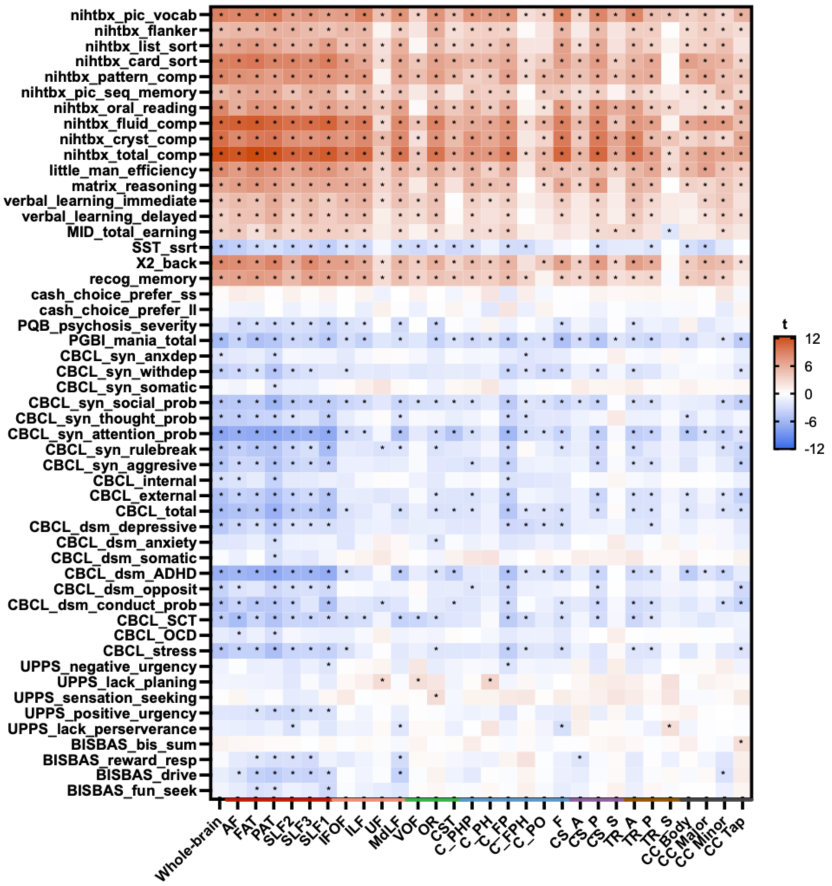


**Supplementary Figure 8. Heatmap of univariate associations between tract-based BAGs (without age-bias correction) and behavioral measures.**

The t-values from the Generalized Linear Model (GLM) analysis are shown. Asterisks indicate two-tailed $p < 0.05$ after False Discovery Rate (FDR) correction. Abbreviation: BAG, Brain Age Gap; AF, Arcuate Fasciculus; FAT, Frontal Aslant Tract; PAT, Parietal Aslant Tract; SLF, Superior Longitudinal Fasciculus; IFOF, Inferior Fronto Occipital Fasciculus; ILF, Inferior Longitudinal Fasciculus; UF, Uncinate Fasciculus; MdLF, Middle Longitudinal Fasciculus; C_FPH, Cingulum Frontal Parahippocampal; C_FP, Cingulum Frontal Parietal; C_PH, Cingulum Parahippocampal; C_PHP, Cingulum Parahippocampal Parietal; C_PO, Cingulum Parolfactory; F, Fornix; CST, Corticospinal Tract; OR, Optic Radiation; VOF, Vertical Occipital Fasciculus; TR_A, Thalamic Radiation Anterior; TR_P, Thalamic Radiation Posterior; TR_S, Thalamic Radiation Superior; CS_A, Corticostriatal Tract Anterior; CS_P, Corticostriatal Tract Posterior; CS_S, Corticostriatal Tract Superior; CC, Corpus Callosum; CC_Body, CC Body; CC_Major, CC Forceps Major; CC_Minor, CC Forceps Minor; CC_Tap, CC Tapetum; nihtbx: NIH Toolbox; MID: Monetary Incentive Delay; SST_ssrt: Stop Signal Reaction Time of Stop Signal Task; PQB: Prodromal Questionnaire Brief Version; PGBI: Parent General Behavior Inventory; CBCL: Children Behavior Check List; ADHD: Attention-deficit/hyperactivity Disorder; SCT: Sluggish Cognitive Tempo; OCD: Obsessive-compulsive Disorder; UPPS: Urgency, Perseverance, Premeditation and Sensation-seeking; BISBAS: Behavioral Inhibition & Behavioral Activation Scales. See Supplementary Table 19 and 20 for more details of the behavioral measures. Source data are provided as a Source Data file.

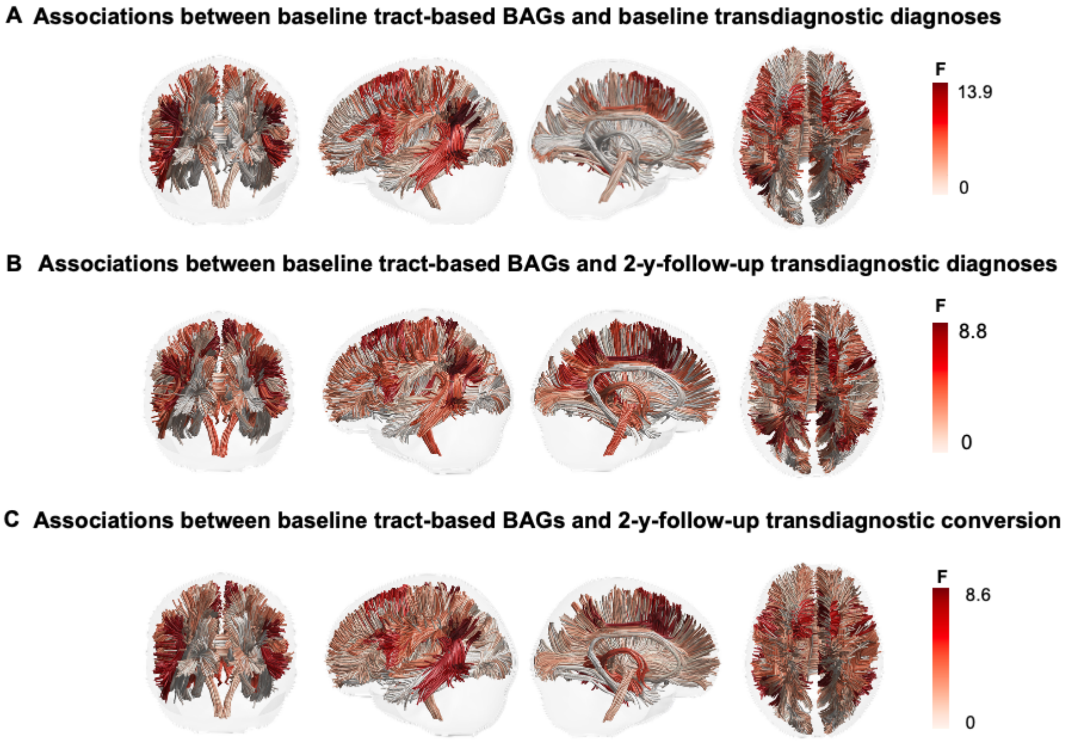


**Supplementary Figure 9. Associations between baseline tract-based brain age gaps and transdiagnostic diagnoses across baseline and 2-year follow-up**

Relationships between baseline tract-BAGs (with age-bias correction) and the cumulative number of KSADS-5 diagnoses at baseline (n = 8,594; panel A), at 2-y-follow-up (n = 7,907; panel B), and the transition of the transdiagnostic status between baseline and 2-y-follow-up (n = 7,803; panel C). In panels A and B, the F values of group-wise KSADS-5 effects for different tracts are plotted. The color of each tract in the upper panel corresponds to the F value of the tract-based BAGs. The panel C displays the specific tract-based BAGs of four groups with different transdiagnostic diagnosis status. Abbreviations: BAG, brain-age gap; KSADS-5: Kiddie Schedule for Affective Disorders and Schizophrenia for DSM-5.

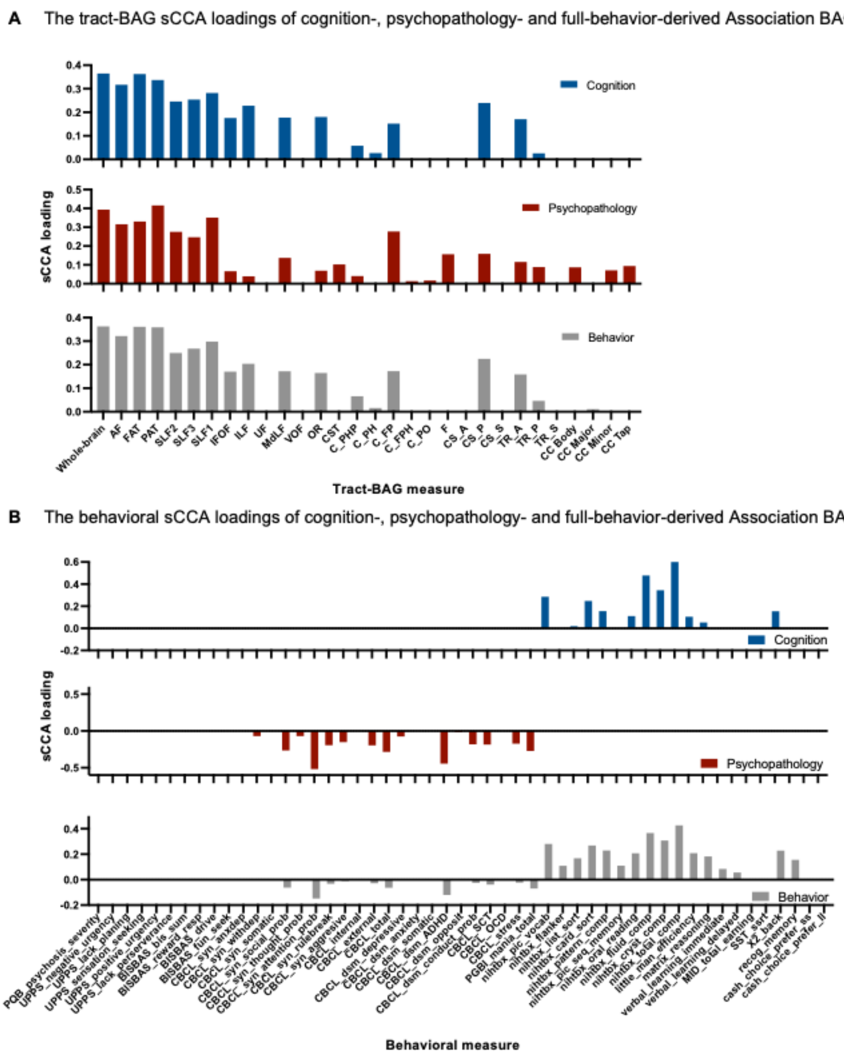


**Supplementary Figure 10. sCCA loadings for tract-based BAG measures and behavioral variables across cognition-, psychopathology-, and full-behavior-derived Association BAG modes.**

(A) Tract-based BAG loadings for the Association BAG mode derived from sCCA variates separately by cognition measures (top, blue), psychopathology measures (middle, red), and the combined behavioral set (bottom, gray). Bars represent feature weights (loadings), indicating the contribution of each tract-based BAG measure to this sCCA variate. Tracts within the dorsal association systems showed the strongest positive contributions across all derived models. (B) Behavioral loadings corresponding to the same cognition-, psychopathology-, and combined behavioral Association BAG modes. These loadings highlight the consistency of tract-based BAG signatures with behavioral dimensions of cognitive function and psychopathology. Abbreviations: sCCA: sparse canonical correlation analysis; BAG, brain-age gap; sparse canonical correlation analysis; AF, Arcuate Fasciculus; FAT, Frontal Aslant Tract; PAT, Parietal Aslant Tract; SLF, Superior Longitudinal Fasciculus; IFOF, Inferior Fronto Occipital Fasciculus; ILF, Inferior Longitudinal Fasciculus; UF, Uncinate Fasciculus; MdLF, Middle Longitudinal Fasciculus; C_FPH, Cingulum Frontal Parahippocampal; C_FP, Cingulum Frontal Parietal; C_PH, Cingulum Parahippocampal; C_PHP, Cingulum Parahippocampal Parietal; C_PO, Cingulum Parolfactory; F, Fornix; CST, Corticospinal Tract; OR, Optic Radiation; VOF, Vertical Occipital Fasciculus; TR_A, Thalamic Radiation Anterior; TR_P, Thalamic Radiation Posterior; TR_S, Thalamic Radiation Superior; CS_A, Corticostriatal Tract Anterior; CS_P, Corticostriatal Tract Posterior; CS_S, Corticostriatal Tract

Superior; CC, Corpus Callosum; CC_Body, CC Body; CC_Major, CC Forceps Major; CC_Minor, CC Forceps Minor; CC_Tap, CC Tapetum; nihtbx: NIH Toolbox; MID: Monetary Incentive Delay; SST_ssrt: Stop Signal Reaction Time of Stop Signal Task; PQB: Prodromal Questionnaire Brief Version; PGBI: Parent General Behavior Inventory; CBCL: Children Behavior Check List; ADHD: Attention-deficit/hyperactivity Disorder; SCT: Sluggish Cognitive Tempo; OCD: Obsessive-compulsive Disorder; UPPS: Urgency, Perseverance, Premeditation and Sensation-seeking; BISBAS: Behavioral Inhibition & Behavioral Activation Scales. See Supplementary Table 19 and 20 for more details of the behavioral measures. Source data are provided as a Source Data file.

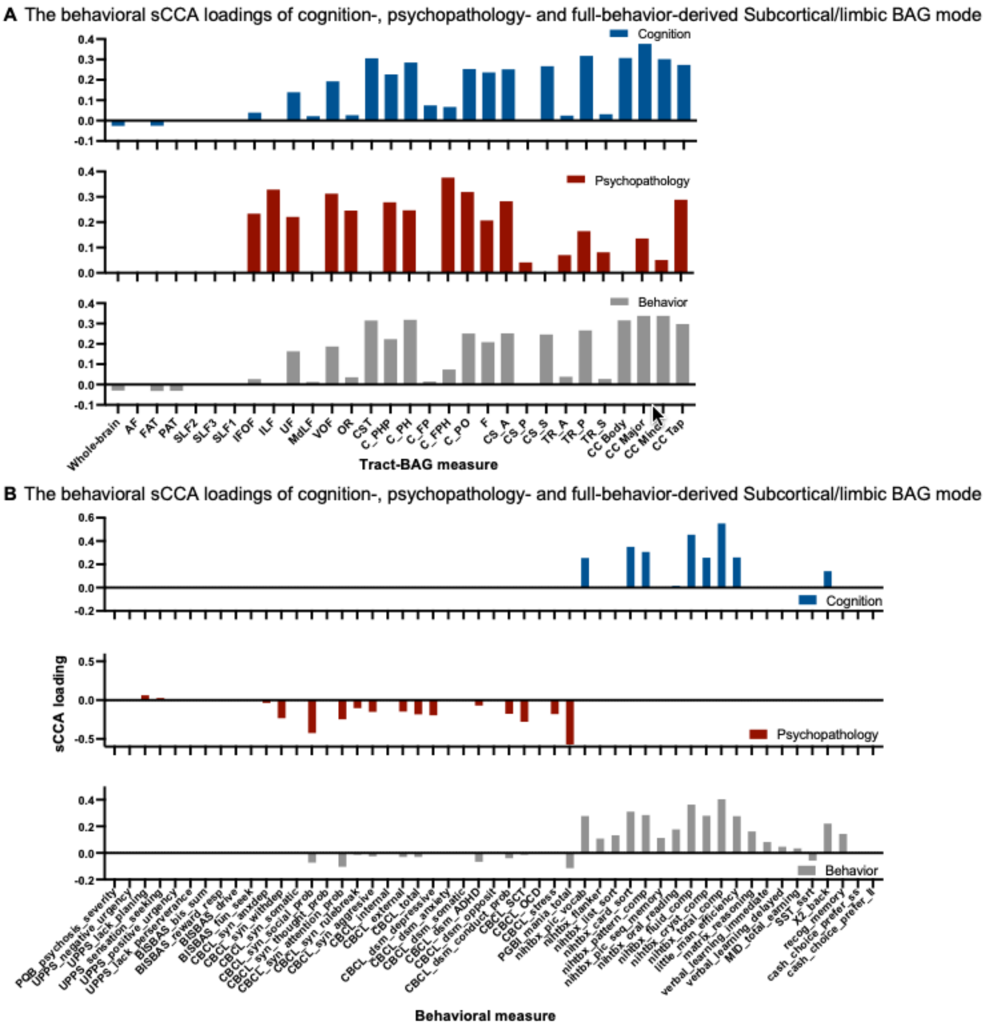


**Supplementary Figure 11. sCCA loadings for tract-based BAG measures and behavioral variables across cognition-, psychopathology-, and behavior-derived Subcortical/limbic BAG modes.**

(A) Tract-based BAG loadings for the Subcortical/limbic BAG mode derived from sCCA variates separately by cognition measures (top, blue), psychopathology measures (middle, red), and the combined behavioral set (bottom, gray). Bars represent feature weights (loadings), indicating the contribution of each tract-based BAG measure to this sCCA variate. Tracts within the limbic or subcortical systems showed the strongest positive contributions across all derived models. (B) Behavioral loadings corresponding to the same cognition-, psychopathology-, and combined behavioral Subcortical/limbic BAG modes. These loadings highlight the consistency of tract-based BAG signatures with behavioral dimensions of cognitive function and psychopathology. Abbreviations: BAG, brain-age gap; sCCA: sparse canonical correlation analysis; AF, Arcuate Fasciculus; FAT, Frontal Aslant Tract; PAT, Parietal Aslant Tract; SLF, Superior Longitudinal Fasciculus; IFOF, Inferior Fronto Occipital Fasciculus; ILF, Inferior Longitudinal Fasciculus; UF, Uncinate Fasciculus; MdLF, Middle Longitudinal Fasciculus; C_FPH, Cingulum Frontal Parahippocampal; C_FP, Cingulum Frontal Parietal; C_PH, Cingulum Parahippocampal; C_PHP, Cingulum Parahippocampal Parietal; C_PO, Cingulum Parolfactory; F, Fornix; CST, Corticospinal Tract; OR, Optic Radiation; VOF, Vertical Occipital Fasciculus; TR_A, Thalamic Radiation Anterior; TR_P, Thalamic Radiation Posterior; TR_S, Thalamic Radiation Superior; CS_A, Corticostriatal Tract Anterior; CS_P, Corticostriatal Tract Posterior; CS_S, Corticostriatal Tract Superior; CC, Corpus Callosum; CC_Body, CC Body; CC_Major, CC Forceps Major; CC_Minor, CC

Forceps Minor; CC_Tap, CC Tapetum; nihtbx: NIH Toolbox; MID: Monetary Incentive Delay; SST_ssrt: Stop Signal Reaction Time of Stop Signal Task; PQB: Prodromal Questionnaire Brief Version; PGBI: Parent General Behavior Inventory; CBCL: Children Behavior Check List; ADHD: Attention-deficit/hyperactivity Disorder; SCT: Sluggish Cognitive Tempo; OCD: Obsessive-compulsive Disorder; UPPS: Urgency, Perseverance, Premeditation and Sensation-seeking; BISBAS: Behavioral Inhibition & Behavioral Activation Scales. See Supplementary Table 19 and 20 for more details of the behavioral measures. Source data are provided as a Source Data file.

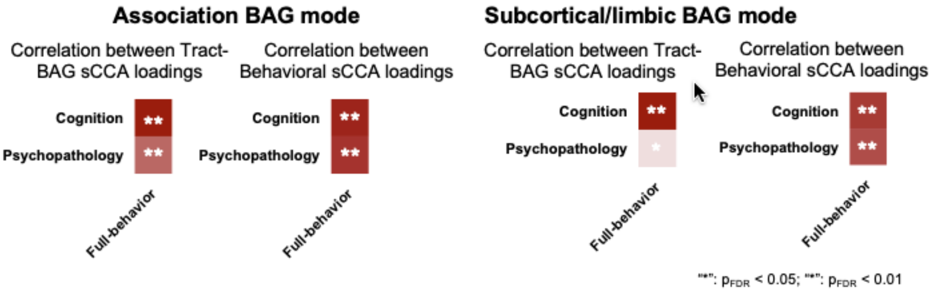


**Supplementary Figure 12. Consistency of tract-BAG and behavioral loading patterns across cognition-, psychopathology-, and full-behavior-derived sCCA modes.**

Heatmaps display Spearman correlation coefficients between loading patterns obtained from sCCA modes derived separately from cognition measures, psychopathology measures, and the full behavioral set. Left panel: Association BAG mode; right panel: Subcortical/limbic BAG mode. More positive correlations indicate more convergent loading patterns across analytic frameworks, highlighting the robustness and shared structure of the Association BAG mode, with less consistent correspondence for the Subcortical/limbic BAG mode. Color bar indicates Spearman correlation coefficient.

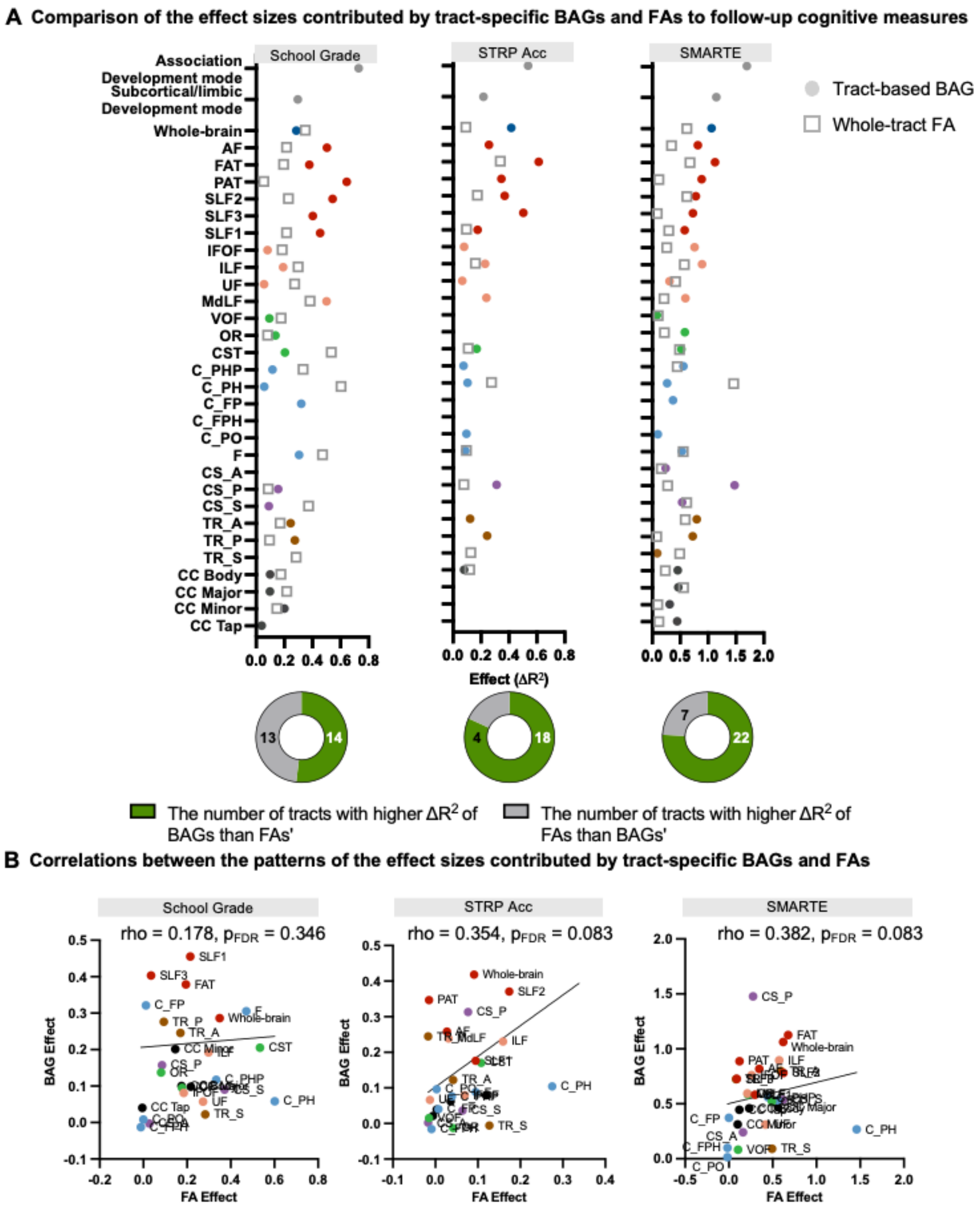


**Supplementary Figure 13. Comparison of tract-specific BAGs and whole-tract FAs in linking to follow-up cognitive performance.**

(A) Effect sizes ($\Delta R^2$) of tract-specific brain age gaps (BAGs; circles) and whole-tract fractional anisotropy (FAs; squares) accounting significant variance for three follow-up cognitive measures: school grade, overall performance of Emotional Stroop Task (STRP Acc), and math ability evaluated by Stanford Mental Arithmetic Response Time Evaluation (SMARTE). Pie charts summarize the number of tracts where BAGs (green) explained more variance than FAs, versus tracts where FAs (gray) explained more variance than BAGs. (B) Scatterplots showing correlations between the effect sizes of BAGs and FAs across tracts for the same three cognitive outcomes. Each dot represents a tract (colored by tract identity), plotted with FA effect on the x-axis and BAG effect on the y-axis. Correlation coefficients (Spearman rho values) and two-tailed p-values indicate the degree of similarity between BAG- and FA-derived effect size patterns across tracts. Abbreviations: BAG: brain age gap; AF, Arcuate Fasciculus; FAT, Frontal Aslant Tract; PAT, Parietal Aslant Tract; SLF, Superior Longitudinal Fasciculus; IFOF, Inferior Fronto Occipital Fasciculus; ILF, Inferior Longitudinal Fasciculus; UF,

Uncinate Fasciculus; MdLF, Middle Longitudinal Fasciculus; C_FPH, Cingulum Frontal Parahippocampal; C_FP, Cingulum Frontal Parietal; C_PH, Cingulum Parahippocampal; C_PHP, Cingulum Parahippocampal Parietal; C_PO, Cingulum Parolfactory; F, Fornix; CST, Corticospinal Tract; OR, Optic Radiation; VOF, Vertical Occipital Fasciculus; TR_A, Thalamic Radiation Anterior; TR_P, Thalamic Radiation Posterior; TR_S, Thalamic Radiation Superior; CS_A, Corticostriatal Tract Anterior; CS_P, Corticostriatal Tract Posterior; CS_S, Corticostriatal Tract Superior; CC, Corpus Callosum; CC_Body, CC Body; CC_Major, CC Forceps Major; CC_Minor, CC Forceps Minor; CC_Tap, CC Tapetum. Source data are provided as a Source Data file.

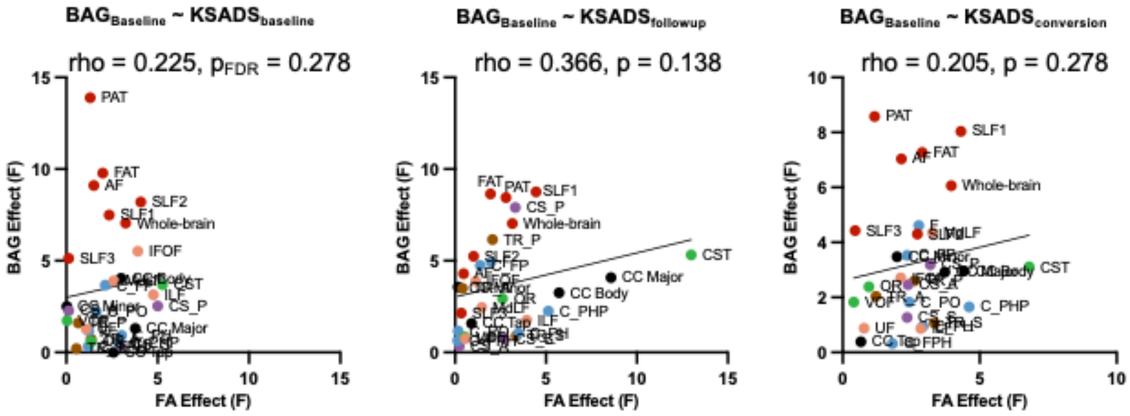


**Supplementary Figure 14. Correlations between tract-specific BAG and FA group with measures related to transdiagnostic psychopathology.**

Scatterplots show the relationships between BAG- and FA-derived effects (y- and x-axes, respectively) for the cumulative number of KSADS-5 diagnoses at baseline (left), at 2-year follow-up (middle), and for conversion status between baseline and 2-year-follow-up (right). Each point represents the F-value of KSADS-5 diagnoses for a tract (colored by tract system). Abbreviations: BAG: Brain age gap; AF, Arcuate Fasciculus; FAT, Frontal Aslant Tract; PAT, Parietal Aslant Tract; SLF, Superior Longitudinal Fasciculus; IFOF, Inferior Fronto Occipital Fasciculus; ILF, Inferior Longitudinal Fasciculus; UF, Uncinate Fasciculus; MdLF, Middle Longitudinal Fasciculus; C_FPH, Cingulum Frontal Parahippocampal; C_FP, Cingulum Frontal Parietal; C_PH, Cingulum Parahippocampal; C_PHP, Cingulum Parahippocampal Parietal; C_PO, Cingulum Parolfactory; F, Fornix; CST, Corticospinal Tract; OR, Optic Radiation; VOF, Vertical Occipital Fasciculus; TR_A, Thalamic Radiation Anterior; TR_P, Thalamic Radiation Posterior; TR_S, Thalamic Radiation Superior; CS_A, Corticostriatal Tract Anterior; CS_P, Corticostriatal Tract Posterior; CS_S, Corticostriatal Tract Superior; CC, Corpus Callosum; CC_Body, CC Body; CC_Major, CC Forceps Major; CC_Minor, CC Forceps Minor; CC_Tap, CC Tapetum; KSADS-5: Kiddie Schedule for Affective Disorders and Schizophrenia for DSM-5 (KSADS-5)

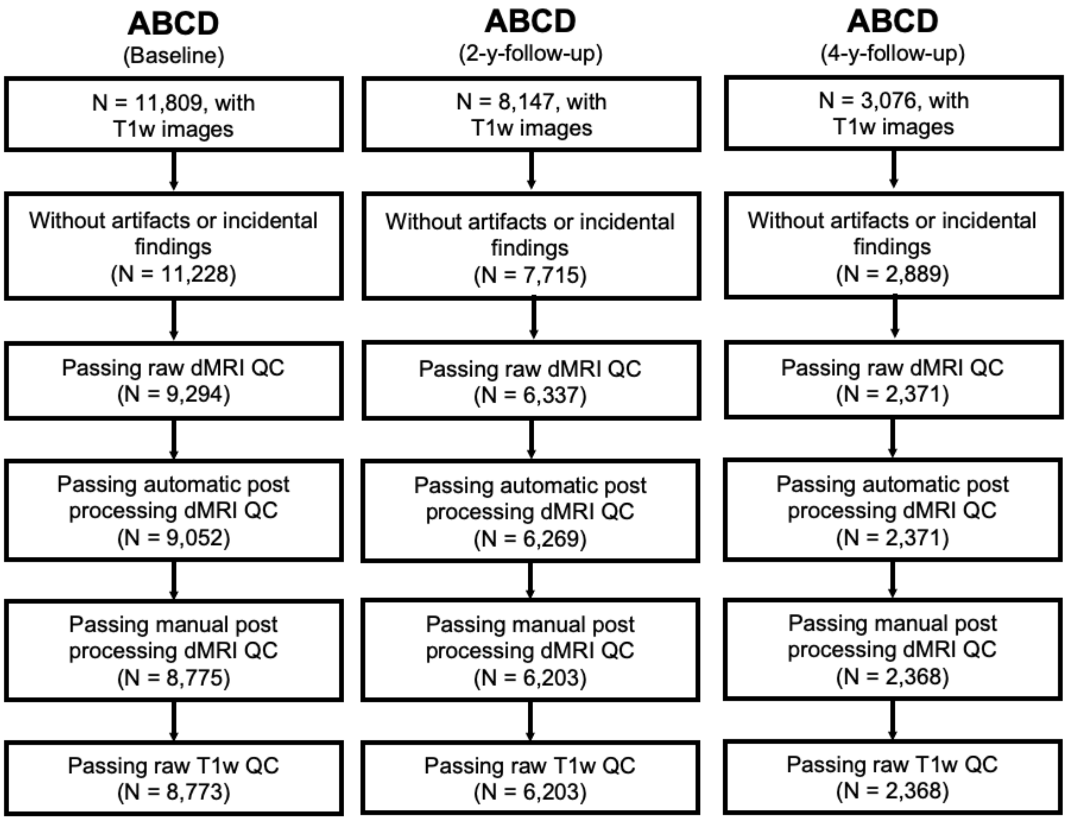


Supplementary Figure 15. **Quality control procedure for the ABCD dataset at baseline, 2-year- and 4-year-follow-ups.**

For each time point, the flowchart shows the number of participants remained after QC steps applied to T1w structural MRI and dMRI data. Abbreviations: QC: quality control; T1w: T1-weighted; dMRI: diffusion MRI; ABCD: Adolescent Brain Cognitive Development.

**Reference**


1. Desikan, R.S., *et al.* An automated labeling system for subdividing the human cerebral cortex on MRI scans into gyral based regions of interest. *Neuroimage* **31**, 968-980 (2006).
2. Luciana, M., *et al.* Adolescent neurocognitive development and impacts of substance use: Overview of the adolescent brain cognitive development (ABCD) baseline neurocognition battery. *Dev Cogn Neurosci* **32**, 67-79 (2018).
3. Rosenberg, M.D., *et al.* Behavioral and Neural Signatures of Working Memory in Childhood. *J Neurosci* **40**, 5090-5104 (2020).
4. Loewy, R.L., Pearson, R., Vinogradov, S., Bearden, C.E. & Cannon, T.D. Psychosis risk screening with the Prodromal Questionnaire — Brief Version (PQ-B). *Schizophrenia Research* **129**, 42-46 (2011).
5. Youngstrom, E.A., Frazier, T.W., Demeter, C., Calabrese, J.R. & Findling, R.L. Developing a 10-item mania scale from the Parent General Behavior Inventory for children and adolescents. *J Clin Psychiatry* **69**, 831-839 (2008).
6. Achenbach, T.M. & Verhulst, F. Achenbach system of empirically based assessment (ASEBA). *Burlington, Vermont* (2010).
7. Zapolski, T.C., Stairs, A.M., Settles, R.F., Combs, J.L. & Smith, G.T. The measurement of dispositions to rash action in children. *Assessment* **17**, 116-125 (2010).
8. Barch, D.M., *et al.* Demographic, physical and mental health assessments in the adolescent brain and cognitive development study: Rationale and description. *Dev Cogn Neurosci* **32**, 55-66 (2018).
9. Pagliaccio, D., *et al.* Revising the BIS/BAS Scale to study development: Measurement invariance and normative effects of age and sex from childhood through adulthood. *Psychol Assess* **28**, 429-442 (2016).
