# Supplementary material for: Deviation in development of dorsal association tracts during preadolescence links to concurrent and future cognitive performance and transdiagnostic psychopathology
Source: Nat Commun. 2026 Feb 19;17:2943. doi: 10.1038/s41467-026-69774-6 (PMC13031853; doi:10.1038/s41467-026-69774-6)
Supplement: Supplementary file 2 — Reporting Summary [file 41467_2026_69774_MOESM2_ESM.pdf]

Corresponding author(s): Yihong Yang

Last updated by author(s): Jan 22, 2026

Reporting Summary

Nature Portfolio wishes to improve the reproducibility of the work that we publish. This form provides structure for consistency and transparency in reporting. For further information on Nature Portfolio policies, see our Editorial Policies and the Editorial Policy Checklist.

Statistics

For all statistical analyses, confirm that the following items are present in the figure legend, table legend, main text, or Methods section.

n/a	Confirmed	
☐	☒	The exact sample size (*n*) for each experimental group/condition, given as a discrete number and unit of measurement
☐	☒	A statement on whether measurements were taken from distinct samples or whether the same sample was measured repeatedly
☐	☒	The statistical test(s) used AND whether they are one- or two-sided<br>*Only common tests should be described solely by name; describe more complex techniques in the Methods section.*
☐	☒	A description of all covariates tested
☐	☒	A description of any assumptions or corrections, such as tests of normality and adjustment for multiple comparisons
☐	☒	A full description of the statistical parameters including central tendency (e.g. means) or other basic estimates (e.g. regression coefficient) AND variation (e.g. standard deviation) or associated estimates of uncertainty (e.g. confidence intervals)
☐	☒	For null hypothesis testing, the test statistic (e.g. *F*, *t*, *r*) with confidence intervals, effect sizes, degrees of freedom and *P* value noted<br>*Give P values as exact values whenever suitable.*
☒	☐	For Bayesian analysis, information on the choice of priors and Markov chain Monte Carlo settings
☒	☐	For hierarchical and complex designs, identification of the appropriate level for tests and full reporting of outcomes
☐	☒	Estimates of effect sizes (e.g. Cohen's *d*, Pearson's *r*), indicating how they were calculated

Our web collection on statistics for biologists contains articles on many of the points above.

Software and code

Policy information about availability of computer code

Data collection	No custom software or code was involved in data collection. Data used in this study are all directly available from ABCD, HCP-D and HBN datasets, which is described in details in the paper.
Data analysis	All data analyses used open-source software and packages:<br>• DSI studio (Chen-2022-12-22): https://hub.docker.com/r/dsistudio/dsistudio/, Automatic tract extraction and tract profile quantification.<br>• ComBat in Matlab 2022a: https://github.com/Jfortin1/ComBatHarmonization/tree/master/Matlab/, harmonization of tract profile data across sites.<br>• Brain age prediction by Gaussian Process Regression (GPR) model in Python 3.10: https://github.com/garedaba/brainAges/<br>• NIMARE: https://nimare.readthedocs.io/en/latest/installation.html/, NeuroSynth decoding.<br>• Brainsmash: https://github.com/murraylab/brainsmash/tree/master/, gray matter voxel-wise spatial permutation for NeuroSynth decoding results.<br>• rotate_parcellation: https://github.com/frantisekvasa/rotate_parcellation/, tract-wise spatial permutation.<br>• Sparse CCA analysis in R 4.5.1: https://github.com/cedricx/sCCA/tree/master/sCCA/code/final/, associations between tract brain age gaps (BAGs) and behavioral assessments.<br>• Generalized linear and generalized additive models relied on R 4.5.1 and the following packages: stats (4.5.1) and mgcv (1.9.3).<br>• Mapping the data onto the brain surface: Connectome Workbench (v2.1.0)

For manuscripts utilizing custom algorithms or software that are central to the research but not yet described in published literature, software must be made available to editors and reviewers. We strongly encourage code deposition in a community repository (e.g. GitHub). See the Nature Portfolio guidelines for submitting code & software for further information.

Data

Policy information about availability of data

All manuscripts must include a data availability statement. This statement should provide the following information, where applicable:

- Accession codes, unique identifiers, or web links for publicly available datasets
- A description of any restrictions on data availability
- For clinical datasets or third party data, please ensure that the statement adheres to our policy

Adolescent Brain Cognitive Development (ABCD) Study® (Release 5.1) is a publicly available dataset accessible via the National Institute of Mental Health (NIMH) Data Archive (NDA): https://nda.nih.gov/abcd. Access can be requested at https://nda.nih.gov/abcd/request-access/.
The Lifespan Human Connectome Project in Development (HCP-D) dataset (https://www.humanconnectome.org/study/hcp-lifespan-development) is also publicly available and can be downloaded via the NDA portal at: https://nda.nih.gov/general-query.html?q=query=featured-datasets:HCP%20Aging%20and%20Development/ with data access. We downloaded the preprocessed SRC files of the HCP-D dataset from the Fiber Data Hub (https://brain.labsolver.org/hcp_d.html) using NDA-provided access.
Developmental neuroimaging data from the Healthy Brain Network (HBN) were obtained from: https://fcon_1000.projects.nitrc.org/indi/cmi_healthy_brain_network/.
In addition, this study utilized publicly available meta-analytic maps from the NeuroSynth database (version 7, https://github.com/neurosynth/neurosynth-data), mitochondrial maps from NeuroVault (https://neurovault.org/collections/16418/), and the Population-Probability Atlas of fiber tracts (https://brain.labsolver.org/hcp_trk_atlas.html).

Research involving human participants, their data, or biological material

Policy information about studies with human participants or human data. See also policy information about sex, gender (identity/presentation), and sexual orientation and race, ethnicity and racism.

Reporting on sex and gender	Throughout this manuscript, we use the term sex to refer to biological sex assigned at birth, as reported via self-administered questionnaires in the HCP-D, HBN, and ABCD datasets. Accordingly, our findings are applicable to both male and female participants. Sex was included as a covariate in all statistical analyses. All participants provided written informed consent for the sharing of individual-level data.<br>For the ABCD dataset, we used data from N = 8,688 participants (4,205 females) at baseline, N = 5,883 participants (2,741 females) at the 2-year follow-up and N = 2,351 participants (1,118 females) at the 4-year follow-up<br>For the HCP-D dataset, we included N = 611 participants (330 females).<br>For the HBN dataset used for testing the brain age prediction performance, we included N = 978 participants (366 females).
Reporting on race, ethnicity, or other socially relevant groupings	Participants' race and ethnicity were reported by their parents. This study did not include race or ethnicity as covariates in statistical models, nor did it use these constructs as proxies for other variables. The distributions of race and ethnicity across datasets are provided below:<br>HCP-D: 63.3% White, 10.7% Black or African American, 7.4% Asian, 15.5% Hispanic, 16.5% Other<br>ABCD: 52.1% White, 15.0% Black or African American, 2.1% Asian, 20.3% Hispanic, 10.5% Other<br>The "Other" category includes participants identifying with multiple races, Native Hawaiian, American Indian or Alaska Native, and Pacific Islander.
Population characteristics	• HCP-D: 611 participants (330 females), ages 5.58 to 21.92 years<br>• HBN: 978 participants (366 females), ages 5.58 to 21.90 years<br>• ABCD: 8,688 participants (4,205 females) at baseline; 5,883 participants (2,741 females) at 2-year follow-up; 2,351 participants (1,118 females) at 4-year-follow-up; age range = 8.91-15.70 years
Recruitment	The HCP-D dataset enrolled healthy children and adolescents across four U.S. sites (University of Minnesota, Harvard University, Washington University in St. Louis, and University of California, Los Angeles), with a recruitment strategy designed to balance age and sex distributions while reflecting the racial and ethnic diversity of the U.S. population. Detailed recruitment criteria are available at Marek et al., 2019: https://doi.org/10.1016/j.neuroimage.2018.08.050/.<br>HBN employed a community-referred recruitment model. The advertisements encouraged the participation of families who have concerns about psychiatric symptoms in their child. Recruitment was coordinated by the Child Mind Institute in New York City. Further details are provided in Alexander et al., 2017: https://www.nature.com/articles/sdata2017181/.<br>The Adolescent Brain Cognitive Development (ABCD) study recruited a large and demographically diverse cohort of preadolescents from 21 sites across the United States. Using probability sampling of public and private elementary schools, the study aimed to construct a sample broadly representative of the U.S. population in terms of race, ethnicity, socioeconomic status, and geographic distribution. Recruitment methods were designed to reduce selection bias and maximize generalizability. A full description of recruitment procedures is provided by Garavan et al. (2018): https://doi.org/10.1016/j.dcn.2018.04.004/.
Ethics oversight	HCP-D: approved by a central Institutional Review Board at Washington University in St. Louis.<br>HBN: approved by the Chesapeake Institutional Review Board.<br>ABCD: approved by the central Institutional Review Board at the University of California, San Diego.

Note that full information on the approval of the study protocol must also be provided in the manuscript.

Field-specific reporting

Please select the one below that is the best fit for your research. If you are not sure, read the appropriate sections before making your selection.

☒ Life sciences ☐ Behavioural & social sciences ☐ Ecological, evolutionary & environmental sciences

For a reference copy of the document with all sections, see nature.com/documents/nr-reporting-summary-flat.pdf

Life sciences study design

All studies must disclose on these points even when the disclosure is negative.

Sample size	This study utilized three large-scale developmental datasets (~10,000 subjects) to assess the reproducibility and generalizability of tract-based brain age prediction models. These three datasets are publicly available and include large developmental samples with high-quality multi-shell dMRI data. The models were trained on the HCP-D cohort and independently validated using the ABCD and HBN datasets, thereby demonstrating the robustness and cross-cohort generalizability of our results. Additionally, the ABCD dataset was used to evaluate the clinical utility of tract-based brain age gaps (BAGs) in relation to cognitive performance and transdiagnostic psychopathology. The sample size was determined on the availability of brain and behavioral measures in these datasets. Our sample size is similar to those reported in previous studies. All participants with quality-controlled neuroimaging and demographic data were included in the analyses.
Data exclusions	For all three developmental datasets, participants were excluded if they lacked demographic information, T1-weighted or diffusion MRI scans, or if their imaging data failed quality control. Details of the image quality control procedures are provided in the Methods section.
Replication	Brain-age prediction: We used a combination of cross-validation and independent testing to assess the reproducibility of tract-based brain age prediction. Within the HCP-D dataset, five-fold cross-validation was used to evaluate model performance. The trained models were then applied to the independent ABCD and HBN datasets. Significant predictive performance was also observed.<br>Relationships between tract-based brain age gaps (BAGs) and transdiagnostic status: To replicate the associations between tract-based BAGs and transdiagnostic psychopathology, we utilized clinical and imaging data from the ABCD study's 2-year follow-up collection. Two Development Modes still showed significant differences across subgroups defined by the cumulative number of psychiatric diagnoses.
Randomization	Randomization was not applicable to this study, as it did not involve separate experimental groups or intervention conditions.
Blinding	All included participants completed the same study protocol. Blinding was not applicable to this study.

Reporting for specific materials, systems and methods

We require information from authors about some types of materials, experimental systems and methods used in many studies. Here, indicate whether each material, system or method listed is relevant to your study. If you are not sure if a list item applies to your research, read the appropriate section before selecting a response.

Materials & experimental systems

n/a	Involved in the study	
☒	☐	Antibodies
☒	☐	Eukaryotic cell lines
☒	☐	Palaeontology and archaeology
☒	☐	Animals and other organisms
☒	☐	Clinical data
☒	☐	Dual use research of concern
☒	☐	Plants

Methods

n/a	Involved in the study	
☒	☐	ChIP-seq
☒	☐	Flow cytometry
☐	☒	MRI-based neuroimaging

Plants

Seed stocks	N/A
Novel plant genotypes	N/A
Authentication	N/A

Magnetic resonance imaging

Experimental design

Design type	N/A
Design specifications	N/A
Behavioral performance measures	No tasks were performed.

Acquisition

Imaging type(s)	Diffusion MRI, T1-weighted MRI and field maps
Field strength	3.0 Tesla
Sequence & imaging parameters	For T1-weighted images:<br>HCP-D: sequence = Multi-echo T1w MPRAGE; TR/TE (ms) = 2500/(1.8/3.6/5.4/7.2); TI (ms) = 1000; Flip angle (°) = 8°; FOV = 256 x 240 x 166; Resolution = 0.8 x 0.8 x 0.8.<br>HBN: sequence = MPRAGE; TR/TE (ms) = 2500/3.15 ; TI (ms) = 1060; Flip angle (°) = 8°; Resolution (mm) = 0.8 x 0.8 x 0.8.<br>ABCD: sequence = MPRAGE; TR/TE (ms) = 2500/2.88[Siemens],2500/2[GE],6.31/2.9[Philips]; Flip angle (°) = 8°; FOV = 256 x 256; Resolution (mm) = 1.0 x 1.0 x 1.0.<br>For diffusion images:<br>HCP-D: multi-shell; sequence = Multiband EPI; b-values = 1,500 and 3,000; 370 directions; no cardiac gating<br>HBN: multi-shell; b-values = 1,000 and 2,000; 128 directions; no cardiac gating<br>ABCD: multi-shell; sequence = HARDI; b-values = 500, 1,000, 2,000 and 3,000; 96 directions; no cardiac gating
Area of acquisition	Whole-brain

Diffusion MRI ☒ Used ☐ Not used

Parameters: See above

Preprocessing

Preprocessing software	HCP-D: This dataset underwent the HCP's minimal preprocessing workflow, including the steps of b0 intensity normalization, and corrections of EPI distortion, eddy-current, head-motion and gradient nonlinearity by FSL.<br>HBN Study: The dMRI data was preprocessed by QSIPrep. Preprocessed 3T dMRI data of 1,769 subjects were downloaded from AWS S3 (link: s3://fcp-indi/data/Projects/HBN/BIDS_curated/derivatives/qsiprep/). Because the dMRI data was collected according to 15 different acquisition parameters, we only included the subjects with the most common dMRI acquisition scheme of 'SOTE_64dir_most_common'.<br>ABCD Study: Standard dMRI preprocessing was performed by the ABCD Data Analysis, Informatics and Resource Center, with the following steps of eddy-current correction, head-motion correction, adjusting diffusion gradients for head motion, robust tensor fitting, correcting B0 distortion and gradient distortion using opposite phase encoding pairs of b0 images, registering b0 images to T1w images using mutual information and cubic interpolation to resample at a 1.7 mm isotropic resolution.
Normalization	Diffusion MRI data were resampled to AC-PC space.
Normalization template	The diffusion data were not analyzed in normalization space.
Noise and artifact removal	Diffusion MRI images were corrected for EPI distortion, eddy-current, head-motion and gradient nonlinearity distortions.
Volume censoring	N/A

Statistical modeling & inference

Model type and settings	• Associations between tract-based BAGs and behavioral assessments<br>We used generalized linear models (GLMs) to examine the associations between tract-based brain age gap (BAG) measures and behavioral scores. In each model, the tract-BAG was treated as the dependent variable, the behavioral score as the independent variable, and age and sex were included as covariates.<br><br>• Associations between tract-based BAGs and clinical diagnoses<br>We applied generalized linear models with tract-BAG measures as the dependent variable and clinical diagnosis (group status) as the independent variable. Age and sex were included as covariates in all models.<br><br>• Associations between baseline tract-based BAGs and follow-up cognitive performance<br>We employed generalized additive models (GAMs) to assess whether baseline tract-BAG measures predicted cognitive performance at follow-up. Tract-BAG was included as the dependent variable, with clinical group as the independent variable. Age and sex were included as covariates.

Effect(s) tested

• Associations between tract-based BAGs and behavioral assessments
The main effects of behavioral measures were evaluated using t-statistics. Corresponding t-values and p-values are reported.
• Associations between tract-based BAGs and clinical diagnoses
The main effects of clinical group were assessed using analysis of variance. F-values and associated p-values are reported.
• Associations between baseline tract-based BAGs and follow-up cognitive performance
The unique contribution of each tract-based BAG measure was quantified by the change in adjusted model variance (ΔR^2, expressed as a percentage) when the BAG predictor was added to a covariate-only (null) model. Both effect sizes (ΔR^2) and p-values are reported.

Specify type of analysis: ☐ Whole brain ☒ ROI-based ☐ Both

Anatomical location(s): Population-Probability Atlas of fiber tracts (https://brain.labsolver.org/hcp_trk_atlas.html)

Statistic type for inference (See Eklund et al. 2016): Voxel-wise or cluster-wise analyses were not conducted.

Correction: False discovery rate (FDR) was applied and described in the methods.

Models & analysis

n/a	Involved in the study
☒	☐ Functional and/or effective connectivity
☒	☐ Graph analysis
☐	☒ Multivariate modeling or predictive analysis

Multivariate modeling and predictive analysis

• Brain age prediction:
Brain age prediction models were trained and validated using the HCP-D dataset, and subsequently applied to independent datasets (ABCD and HBN) to assess generalizability. Specifically, we implemented five-fold cross-validation on 80% of the HCP-D participants using Gaussian Process Regression to predict chronological age based on either whole-brain tract profiles or 29 tract-specific profiles. The remaining 20% of HCP-D participants were used for validation within each fold. Trained models were then applied to predict brain age in both the ABCD dataset (at baseline and 2-year follow-up) and the HBN dataset. Model accuracy was evaluated in the cross-validation dataset and the independent testing dataset using the R2 (the portion of the variance explained) and the MAE (mean absolute error).
• Sparse canonical correlation analysis:
sCCA was used to identify latent dimensions that maximally correlate brain features with behavioral measures. Regularization parameters were optimized via grid search by repeatedly sampling two-thirds of the subjects across ten iterations. The parameter set yielding the highest canonical correlation was selected for the final model. Statistical significance of each canonical mode was assessed using 1,000 permutation tests, in which the order of the behavioral data was shuffled. To evaluate the significance of individual behavioral variables within each mode, we conducted 1,000 bootstrap resamples. Behavioral variables were considered significant if their 95% confidence intervals excluded zero.
